# Prospective pilot safety, feasibility study of an optic-to-audio device for children with CLN3 disease

**DOI:** 10.1186/s13023-026-04319-0

**Published:** 2026-04-03

**Authors:** Thuy Tien Nguyen, Andrea Munoz, Kisha Jenkins, Ibukun Agbede, Wadih M. Zein, Laryssa A. Huryn, Brian P. Brooks, Jennifer Chisholm, Julie Christensen, Christopher Zalewski, M. Teresa Magone, Colby Chlebowski, Audrey Thurm, An N. Dang Do

**Affiliations:** 1 Occupational Therapy Service, Mark O. Hatfield Clinical Center, National Institutes of Health, Bethesda, MD USA; 2 Eunice Kennedy Shriver National Institute of Child Health and Human Development, National Institutes of Health, Bethesda, MD USA; 3 Ophthalmic Genetics and Visual Function Branch, National Eye Institute, National Institutes of Health, Bethesda, MD USA; 4 Auditory and Vestibular Clinical Research Section, National Institute on Deafness and Communication Disorders, National Institutes of Health, Bethesda, MD USA; 5 Consult Services Section, National Eye Institute, National Institutes of Health, Bethesda, MD USA; 6 National Institute of Mental Health, National Institutes of Health, Bethesda, MD USA; 7 Unit on Cellular Stress in Development and Diseases, Eunice Kennedy Shriver National Institute of Health and Human Development, National Institutes of Health, 10 Center Drive, Bethesda, MD 20892-1103 USA

**Keywords:** Juvenile batten, Low vision, Assistive device, Patient-reported outcomes

## Abstract

**Background:**

Low-vision rehabilitative support for children with multiple-disability conditions is underexplored. We conduct a pilot study of an assistive device in children with CLN3 disease, a multisystemic pediatric blindness and neurodegenerative condition. The prospective, pilot study (NCT04974307) evaluated the safety, feasibility and preliminary efficacy of the OrCam MyEye 2 in aiding daily living tasks. We used multimodal assessments of feasibility and efficacy to assess nine participants with CLN3 disease and one with non-CLN3 low vision (age 8.1-17.4 years; females:males 2:8) at baseline (Day 1, without the device) and Day 5 (with the device).

**Results:**

Surveyed parents and affected children reported desire for future research addressing the vision loss in CLN3 disease, and lower quality of life in vision-related domains. One grade 1 adverse event was recorded during the study. Feasibility and Function assessments showed that > 90% of evaluable participants scored above thresholds on feasibility assessments and > 25% had improved scores and performance time on device-specific tasks.

**Conclusions:**

The data provide indications for the safety, feasibility and task-specific efficacy for the OrCam MyEye 2. They support further, including longitudinal, evaluation and consideration of such optic-to-audio devices as part of the toolbox for low vision therapy for children with multiple disabilities such as CLN3 disease.

**Trial registration:**

clinicaltrials.gov, NCT04974307. Registered 23 July 2021, https//clinicaltrials.gov/study/NCT04974307.

**Supplementary Information:**

The online version contains supplementary material available at 10.1186/s13023-026-04319-0.

## Background

Comorbidities or multiple disabilities are present at high frequencies in children with visual impairment and is estimated to be as high as 70% in the United Kingdom cohorts [1–3]. The non-ophthalmic, frequently involving global developmental delays [3], component is associated with conditions ranging from those with perinatal (e.g., retinopathy of prematurity, hypoxic or ischemic encephalopathy) to those with genetic or neurologic (e.g., oculocutaneous albinism, neurodegenerative disorders) etiologies [1]. Research into assistive and augmentative approaches for supporting visual impairment in children with multiple disabilities is limited. Motivated by parental feedback and leveraging a prospective and ongoing natural history study of individuals with CLN3 disease, we conducted a pilot study of a potential assistive device for children with this condition.

Juvenile-onset CLN3 or Batten (OMIM# 204200), a recessively inherited, pediatric blindness and neurodegenerative condition with an estimated 1:100,000 prevalence involves biallelic variants in the *CLN3* gene. Progressive visual impairment is an early presentation in over 80% of CLN3 disease cases [4, 5], followed by mental and motor deterioration, epileptic seizures and premature death [6–8]. Signs and symptoms typically present around pre-kindergarten ages. Life expectancy is into the second to third decade of life. There is currently no approved disease-altering therapy for CLN3 disease.

Visual impairment typically begins around 4–7 years of age with progression to legal blindness within 2–4 years [9]. Presentations that often lead to an ophthalmic evaluation include nystagmus, loss of visual acuity, photophobia and loss of peripheral and color vision [10]. Ophthalmic findings include macular degeneration (e.g., bulls-eye maculopathy), retinal pigment accumulation, retinal vascular attenuation, optic nerve atrophy, abnormal electroretinogram responses, and thinning on optical coherence tomography [4, 10, 11]. Low-vision accommodations such as Braille or sign language and assistive or vision-requiring augmentative communication devices have limited duration of usefulness given the neurodegenerative progress of CLN3 disease and its co-morbidities [12] (unpublished observations). Children with the typical CLN3 disease manifestation experience plateauing or decline in neurodevelopmental abilities starting around 7–9 years of age [13–15], behavioral dysregulations that may include anxiety and perseveration peaking in the pre-teen years [13], and onset of seizures and motor dysfunctions during the pre- to early teen years [9, 16, 17]. Additional assistive technology options would be useful in optimizing sensory, cognitive, and motor function while enhancing the ability of children with CLN3 disease to participate in school and home activities.

The OrCam MyEye 2 is a portable, optic-to-audio assistive vision device, advertised to function using simple operational procedures. A pilot study using the OrCam MyEye 2 in 12 adults with low vision and intact cognition showed improved ability in reading and daily function tasks, as well as the device being easy to use [18]. While anecdotal reports of the use of the device in children with low vision are available [19, 20], no formal study has been done to evaluate the OrCam MyEye 2 in children with low vision. We conducted a prospective pilot study of the OrCam MyEye2 in children with CLN3 disease, a pediatric blindness and multiple-disability condition, to evaluate its safety, feasibility, and applicability.

## Participants, materials and methods

### Ethics and study eligibility

The National Institutes of Health Institutional Review Board approved the protocols, conducted in accordance with International Conference on Harmonisation Good Clinical Practice. For the device study (NCT04974307), we enrolled participants 6–18 years of age who had CLN3 disease, an estimated visual acuity in the better seeing eye < 20/200, and an appropriate cognitive developmental ability to participate based on clinical judgment of the study team, using neurodevelopmental or clinical assessment of the participant’s ability to follow verbal instructions. For comparison, children 6–18 years of age with comparable low vision, not related to CLN3 disease, and who had cognitive developmental ability determined by clinical judgment of the study team to allow for participant to follow verbal instructions, were also eligible. Participants who had hearing loss precluding the use of the device, prior usage of an optic-to-audio assistive device, or inability to comply would be excluded. Eligibility criteria for the natural history study (NCT03307304) have been described previously [17]. Appropriate consents or assents were obtained.

### Testing and training

**Fig. 1:**
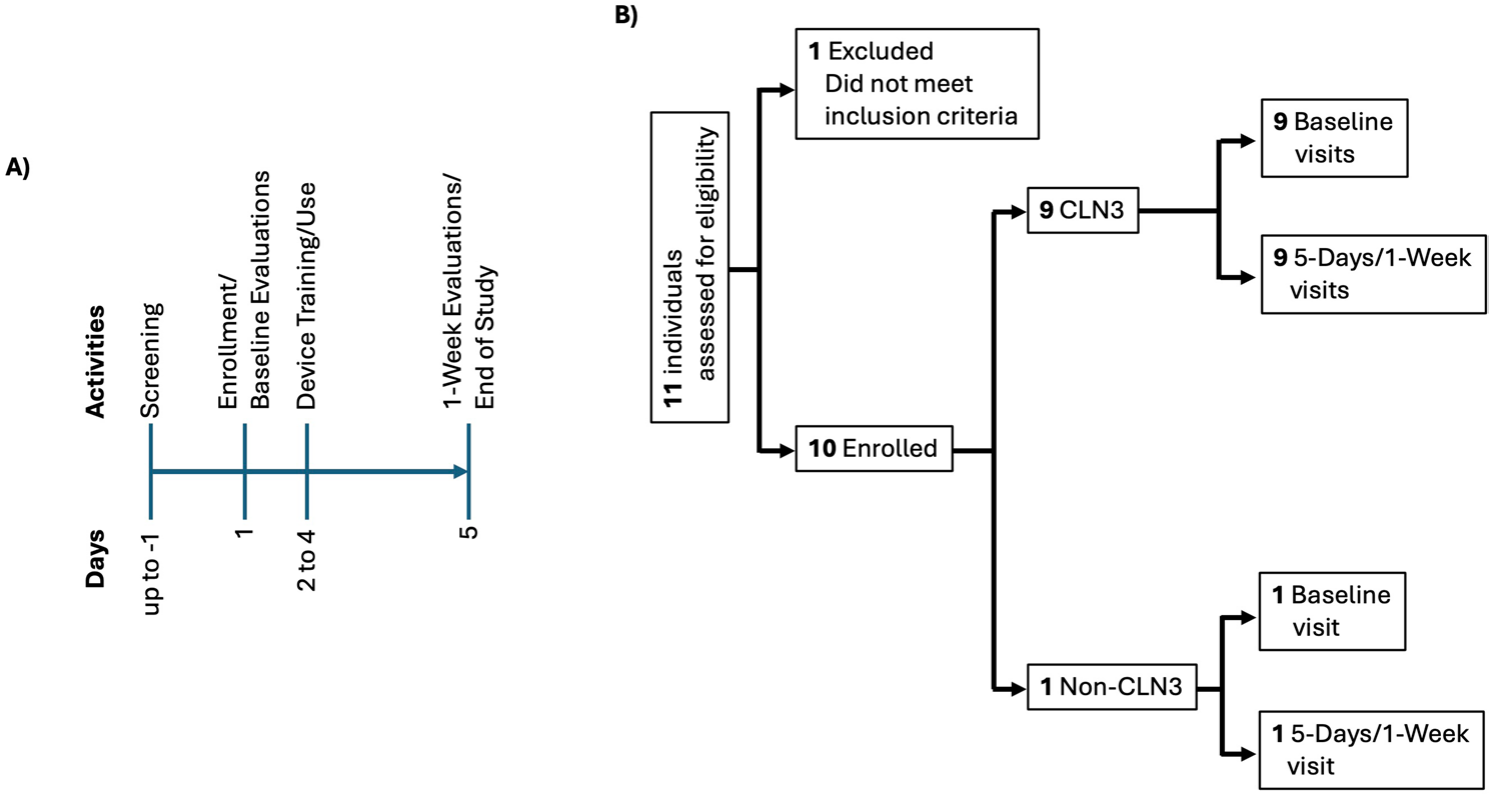
Main study design. **(A)** Study schema. **(B)** Participant recruitment, enrollment, and evaluations

Upon enrollment, participants underwent Day 1 testing, without the use of the device, (Fig. 1A, Additional Files 1, 2). Following the baseline evaluations, participants and parents received training on the device and its use per standardized procedures (Additional File 3). They then continued to receive daily training, and practiced with the device for two days outside of structured study visits (i.e., at home or temporary lodging) (Fig. 1A). Appropriate language and translator were programmed and used for non-English speaking participants. On Day 5, the participants were again tested, this time with the use of the device. The main study and data collection encompassed one week (Fig. 1A). Participants and families were provided an option to participate for an additional one-month of using the device in their home setting (Additional File 4A). The study team conducted weekly check-ins for adverse events and trouble-shooting if needed. At the end of 1-month, participants who elected to do the optional study extension were again tested with the use of the device.

### Study-specific instruments

#### OrCam MyEye 2

The OrCam MyEye 2 is an optic-to-audio assistive vision device that is wearable (Additional File 5). The eyeglass-mounted device is advertised to have text read to the users, and to help users identify faces, objects and colors through audio output by pointing and aiming the camera at the intended target [21]. 

#### Feasibility test

From the manufacturer’s training resources and the study team’s experience in learning how to use the device, we identified main basic actions for which a child with CLN3 disease would need in order to use the OrCam MyEye2 (Additional File 3). The six main basic actions evaluated included abilities to maintain wearing of eyeglass with the device attached, maintain head position looking straight ahead for 10 s, position pointer finger in front of face, maintain correct positioning of pointer finger for 10 s, raise and correctly position hand in front of face and hold for 10 s, and tap the device at the correct location. From these, we developed a 9-item test to evaluate the ease of performing the basic actions (Additional File 1). Trained study team members administered the assessment of feasibility separately at visit Day 1, or in combination with the Function Test (see below; this was done while the participants used the device) at visit Day 5 or optional testing at Day 33 (Additional File 2). Each item was scored as 0/No (could not perform task) or 1/Yes (could perform task). We analyzed the score consisting of the sum of the scores from the first six items. Items 7–9 were exploratory and thus were not included in the total score.

#### Function test

We developed a 10-item, two levels (A and B) per item, assessment to evaluate the ability of the participants to complete tasks executed with the device (Additional File 2). These task categories included recognition of printed text [Items 1,2,5: small font (sans serif, 12–14 point). Items 3,4: large font (sans serif, 72 point)], identification of primary colors (Item 6), recognition of faces (Item 7), reporting date and time (Item 8), recognizing product labels (Item 9), and recognition of screen text (Item 10). Based on participants’ age group, our observations of participants with CLN3 disease in the natural history study (NCT03307304), and parental reports, we designated the first three task categories as constituting core test items 1–7. The remaining exploratory test items 8–10 were not administered to all participants. Test items 1–2,4–6 were administered in-person for visits Days 1 and 5, or remotely for participants who did the optional 1-month extension. Trained study team members administered the test at visit Day 1 without the device, and at subsequent visits with the device. Each item was scored as 0/No (could not perform task) or 1/Yes (could correctly perform task). Time to complete the task correctly was also recorded. If the participant did not correctly complete level A of the test item, testing for level B items was not administered and completion times for both levels were scored as 180 s (the time to cycle through all the test item instructions).

### Study-specific questionnaires

#### Applicability questionnaire

We designed the Applicability Questionnaire (Additional File 6) 10-item Part A for parental rating of how often the child encountered a specific activity (0 = did not encounter, 4 = encountered daily), and 10-item Part B of how helpful a visual assistive device would be in helping the child to complete the specific activity (0 = not helpful, 4 = helpful, parent would not have to help with task). Parents were also asked two open-ended questions about what daily function task(s) the child would be able to do with minimal help if a device such as OrCam MyEye 2 were available, and what capability a device should develop that would be more helpful for the child. Questionnaire was administered at Day 1. Item-level frequencies of ratings were tabulated.

#### Ability questionnaire

We designed an Ability Questionnaire of 15 items for parents (Additional File 7) to rate their child’s ability to perform device-specific tasks from 0 to 4 (0 = unable to or does not understand task; 4 = understands and able to complete task mostly if not all independently). Questionnaire was administered at Days 1, 5, and 33. Item-level scores were compared across visits. 

#### Feasibility questionnaire

We designed the Feasibility Questionnaire (Additional File 8) as 11 binary-response (0/No, 1/Yes) parental questions to evaluate device usability for children with or without CLN3 disease. Questionnaire was administered at Days 5 and 33. Item-level frequencies of ratings were tabulated.

### Validated questionnaires

#### Low vision quality of life (LVQoL)

The LVQoL evaluates the impact of vision loss on activities of daily living in adults with low vision [22]. Its 25 items assess (1) distance vision, mobility, and lighting; (2) adjustment; (3) reading and fine work; (4) activities of daily living. Participants rate each item from 1 (significant issue) to 5 (no issue). We administered the survey on Days 1 and 33. Sum total scores as percentages of maximum possible scores were calculated.

#### Pediatric eye questionnaires (PedEyeQ)

The PedEyeQ evaluates quality of life in children with low vision [23]. The children direct and by proxy surveys contain sub-domains on Functional Vision, Bothered by Vision, Social, Frustration/Worry, and Eyecare. The parent survey queries about Impact on Family, Worry about Eye Condition, Interactions, and Functional Vision. We administered the survey on Days 1 and 33. Rasch scores were obtained, wherein lower scores indicated worse quality of life or functional vision https://public.jaeb.org/pedig/view/reference#pedeyeq (Accessed 2024Jul12).

### Statistical plans and analyses

Data were summarized in Excel and R using descriptive statistics or qualitatively as applicable. For comparisons of before and after device intervention in the same participant and correlation calculations, statistical significance was determined by corresponding non-parametric (paired Wilcoxon Rank sum test) methods. A *p* < 0.05 would be considered significant.

For analyses of the primary endpoints, interpretation of results is rule-based as pre-defined in the study protocol. For the Feasibility Test, device use is feasible if at baseline or Day 5 50% or more of total number of participants score three or greater out of five. For the Feasibility Questionnaire, device use is feasible if at Day 5 50% or more of total number of participants score six or greater out of 11. Analysis of the optional one-month (Day 33) data followed the same rule-based interpretations.

## Results

At their initial CLN3 natural history study (NCT03307304) visit, parents of affected children with CLN3 disease [*N* = 34. Age (years) mean = 11.6, SD = 5.4. Females:Males 17:17] rank ordered (1 = most desirable) the symptoms they would like to see being addressed by future interventions. Fifty percent of the affected individuals have CLN3 disease stage 1 (Additional File 9A, where stage 0 = pre-symptomatic, 1 = vision loss as the only manifestation, 2 = seizures in addition to vision loss, 3 = unable to walk without assistance in addition to manifestations of the preceding stages) [24]. Vision was the second highest choice, with 26% of mothers (6/23) and 31% of fathers (5/16) choosing vision as number one (Additional File 9B). Of all the parental rank orders, vision was tied as the most frequently chosen symptom, with 85% (23/27) of the vision rankings being in the top 3 (Additional File 9C).

From September 2021 to October 2023, we screened 11 individuals, excluded one for having an uncorrected visual acuity better than 20/200, and enrolled 1 individual without and 9 with CLN3 disease (Fig. 1B). The individual without CLN3 disease was referred from the National Eye Institute Consult Clinic with a diagnosis of Hereditary Benign Intraepithelial Dyskeratosis. All 10 participants underwent in-person assessment on Days 1 and 5; three completed the optional 1-month study and Day 33 study evaluations (Additional File 4B). We terminated the study early because of slow recruitment.

The participants aged 8.1–17.4 years (median 10.75, IQR 9.25–13.8) were predominantly males (8/10), with 7/10 speaking English as their primary language (Table 1). Participants’ visual acuity, hearing, neurodevelopmental characteristics, and CLN3 disease staging are in Table 1.

**Table 1:** Demographic and clinical information of study participants

Participant ID	Phenotype	Age (years)	Sex	Race	Primary Language	Visual Acuity		Hearing	Neurodevelopment		CLN3 Stage
						OD	OS		VCI	Vineland ABC	
CAM001	CLN3	12.6	M	Asian	English	CF@6in	CF@6in	Normal	62	64	2
CAM002	CLN3	8.1	M	White	English	LP	LP	Normal	50th centile	79	2
CAM003	Non-CLN3	15.2	M	White	English	CF@1ft	CF@1ft	b/l SNHL corrected with hearing aids	ND	Clinical assessment	Not applicable
CAM004	CLN3	14.2	F	White	English	LP&P	LP&P	Normal	78	68	2
CAM005	CLN3	17.4	M	White	English	CF	CF	Normal	87	74	2
CAM006	CLN3	9.1	M	White	Brazilian Portuguese	LP&P	HM	Normal	ND	87	1
CAM007	CLN3	11.8	M	Asian	English	LPscP	LP&P	Normal	70	44	2
CAM009	CLN3	9.7	M	White	English	LPscP	LPscP	Normal	86	65	1
CAM010	CLN3	9.7	F	White	Spanish	CF	CF	Normal	ND	94	1
CAM011	CLN3	8.1	M	White	Spanish	HM	HM	Normal	ND	Clinical assessment	1

CLN3: syndromic disease presentation. Non-CLN3: hereditary benign intraepithelial dyskeratosis. Age: at enrollment. Visual acuity: best corrected/ obtainable. VCI: verbal comprehension index, 5th edition standard score. Vineland ABC: 3rd edition, adaptive behavior composite standard score. CLN3 disease staging based on Masten et al.: 0 = genetic confirmation; 1 = vision loss; 2 = one or more seizures; 3 = unable to walk without assistance. CF@: counting finger at indicated distance. F: female. HM: hand motion. LP&P: light perception and projection. LPscP: light perception without projection. M: male. ND: not done as deemed not clinically needed or since English is not the primary spoken language. OD: right eye. OS: left eye

### Quality of life and applicability

**Fig. 2:**
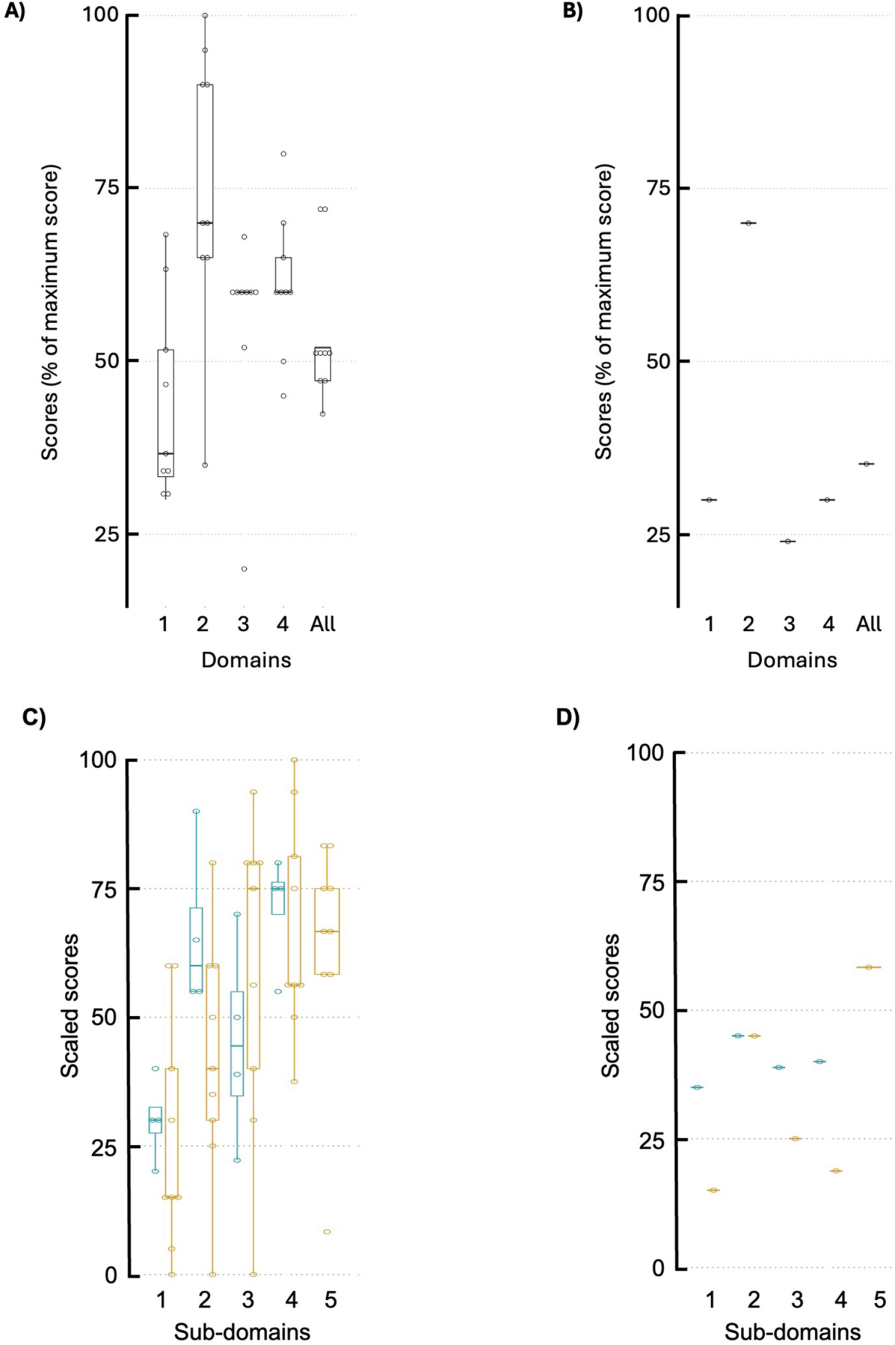
Participant and parental perception of quality of life. Low vision quality of life scores as percent of maximum possible score (**A**,** B**). Domain 1 = distance vision, mobility, lighting. 2 = adjustment. 3 = reading and fine work. 4 = activities of daily living. All=combination of all 4 domains. Participants with CLN3 disease (*n* = 9, **A**). Participant without CLN3 disease (*n* = 1, **B**). PedEyeQ scaled scores of participants’ and proxy caregivers’ perspectives at baseline visit (**C**,** D**). Sub-domain 1 = functional vision. 2 = bothered by vision. 3 = social. 4 = frustration/worry. 5 = eyecare. Horizontal bar: median. Box: interquartile range. Whiskers: 1.5x IQR. Participants with CLN3 disease (*n* = 4; blue) and proxy caregivers (*n* = 9; gold) (**C**). Participant without CLN3 disease (*n* = 1; blue) and proxy caregiver (*n* = 1; gold) (**D**)

Caregivers for individuals with CLN3 disease (*n* = 9) reported sum scores of median = 52; IQR = 47.2,52 on the LVQoL (Fig. 2A). Compared to the maximum possible, scores for individuals with CLN3 disease were also lower in the subdomains of distance vision (median = 37; IQR = 33,52), reading (60; 60,60), and daily living (60; 60,65). The subdomain of adjustment had large variability (70; 65,90). In the single non-CLN3 participant, the score pattern was similar, with the reading subdomain having the lowest score (24%) (Fig. 2B).

In the PedEyeQ assessments, lower scores indicated worse status. At Day 1, individuals with CLN3 disease (*n* = 4) rated the Functional Vision subdomain lowest (median = 30.0; IQR = 27.5,32.5) (Fig. 2C). While they scored higher in the subdomains of Frustrations (44.4; 34.7,55.0), Bothered (60.0; 55.0,71.3), and Social (75.0; 70.0,76.2), the median and IQR scores were 75 or less. Ratings of these subdomains by a proxy caregiver (*n* = 9) provided similar patterns. The participant with non-CLN3 low vision and the proxy reported generally lower scores in these domains (Fig. 2D). From the parental perspective (*n* = 10), the impacts of the child’s low vision were reflected in lower scores across all four subdomains, with the more significantly affected being greater worry about the child’s Condition (32.5; 21.3,35.0) and Functional Vision (28.1; 20.3,40.6) (Additional File 10).

**Fig. 3:**
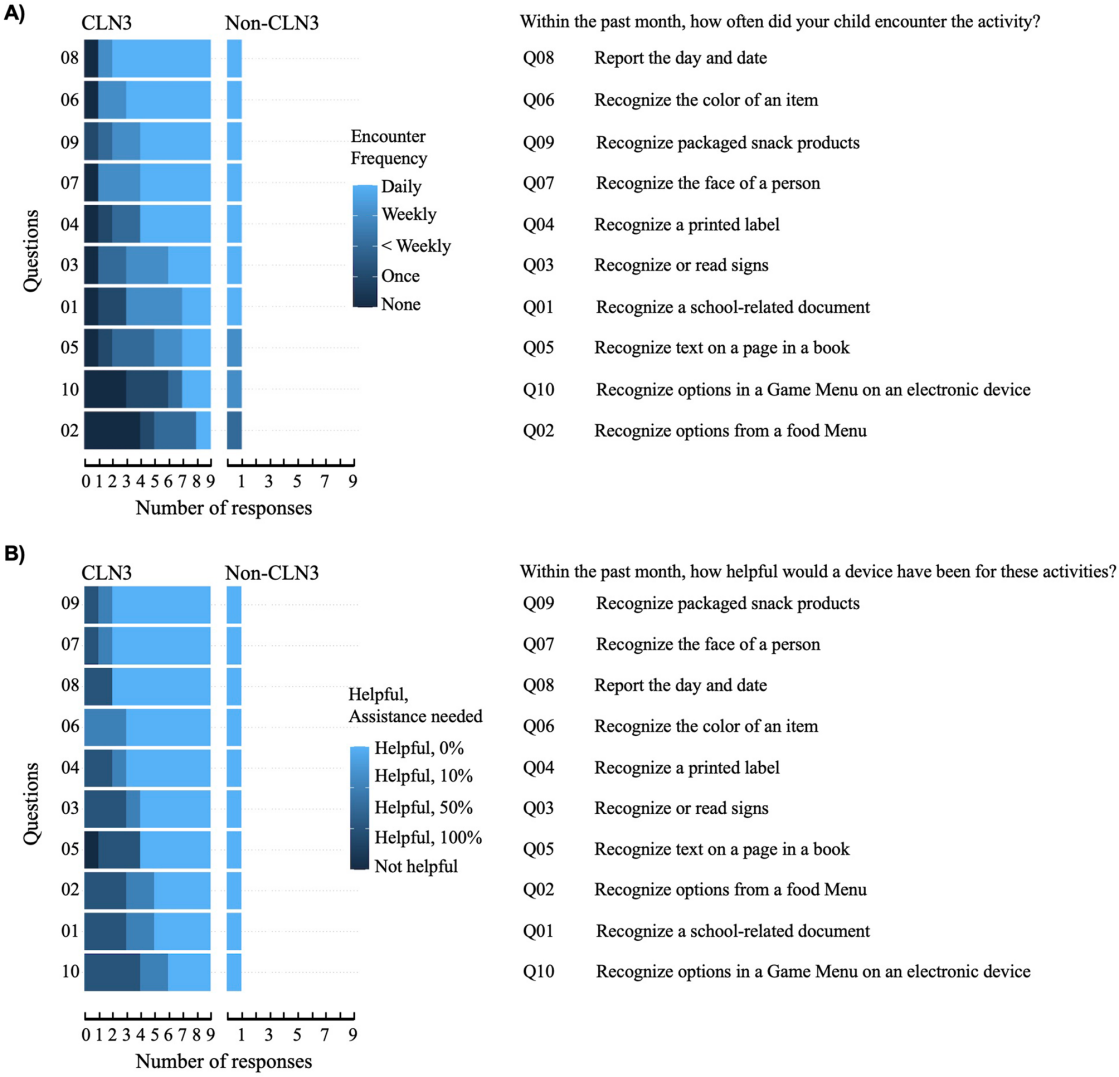
Parental perception of the device applicability. Encounter frequencies of activities performed by the device (**A**). Rating of usefulness of the device in helping with stated activities (**B**). CLN3, *n* = 9. Non-CLN3, *n* = 1

More than half of the parents of children with CLN3 disease (6/9) reported daily encounter with tasks including reporting the day and date, and recognizing colors, packaged snack products, face of a person, or a printed label (Fig. 3A). Rankings from the parent of the child without CLN3 disease were similar (Fig. 3A). Parents responded that the device’s advertised functional capabilities would be helpful (CLN3 *n* = 6/9; non-CLN3 *n* = 1/1) and may help their child perform many of these tasks independently. Notably, small-font text recognition tasks were ranked as those needing additional parental help (Fig. 3B).

### Device tests

#### Safety and feasibility

One grade 1 adverse event related to ear and nose itching while wearing the device and eyeglasses was documented. No other major or minor adverse event was reported. Ninety to 100% of participants successfully performed the 6-item Feasibility Test (Table 2A), indicating participants’ ability to perform specific task required for the use of the device. In four participants, feasibility assessment was done only as part of the Function Test (Table 2B). Feasibility assessed while participants performed the Function Test revealed information on the level of prompting needed. The level of prompting provides additional indicators of ease or feasibility of using the device, where 1 = verbal reminders, 2 = verbal reminder and specific corrective instruction, and 3 = verbal and non-verbal reminder and specific instruction (Additional File 3). At visit Day 5, for text recognition tasks 1A, 2A, 3A and facial recognition task 7A, 2–3 CLN3 participants required level 1 prompting to complete the task. For text recognition tasks 3A and 5A, 1–2 CLN3 participants required level 2 prompting to complete the task. For color recognition task 6A, three CLN3 and the non-CLN3 participants required level 1, and one CLN3 participant did not complete the task despite level 3 prompting.

**Table 2:** Evaluation of feasibility of using the device by all study participants as measured by either **(A)** Feasibility Test at visits Day 1, 5, and 33 or **(B)** Feasibility assessment from Function Test questions 1–7A at visits Day 5, and 33

A)						
Test Items^a^	Number of Participants (CLN3/non-CLN3), *n*					
	Evaluated			Able to Perform Item		
	Day 1	Day 5^b^	Day 33	Day 1	Day 5	Day 33
Maintain wearing eyeglasses	9/1	5/1	2/1	9/1	5/1	2/1
Maintain looking straight ahead	9/1	5/1	2/1	9/1	5/1	2/1
Position pointer finger	9/1	5/1	2/1	8/1	5/1	2/1
Maintain positioning of pointer finger	9/1	5/1	2/1	9/1	5/1	2/1
Raise hand and hold	9/1	5/1	2/1	9/1	5/1	2/1
Tap device	9/1	5/1	2/1	9/1	5/1	2/1

B)						
Test Items^a^	Number of Participants (CLN3/non-CLN3), *n*					
	Evaluated			Able to Perform Item		
Task 1A	Day 5		Day 33	Day 5		Day 33
Aim device towards object	9/1		2/1	9/1		2/1
Maintain head/finger position	9/1		2/1	9/1		2/1
Remove finger on hearing device’s signal	2/0		0/0	2/0		0/0
Complete task without level 1 prompt	9/1		2/1	7/1		2/1
Complete task without level 2 prompt	9/1		2/1	9/1		2/1
Complete task without level 3 prompt	9/1		2/1	9/1		2/1
Task 2A	Day 5		Day 33	Day 5		Day 33
Aim device towards object	9/1		2/1	9/1		2/1
Maintain head/finger position	9/1		2/1	9/1		2/1
Remove finger on hearing device’s signal	2/0		0	2/0		0
Complete task without level 1 prompt	9/1		2/1	7/1		1/0
Complete task without level 2 prompt	9/1		2/1	9/1		2/1
Complete task without level 3 prompt	9/1		2/1	9/1		2/1
Task 3A	Day 5		Day 33	Day 5		Day 33
Aim device towards object	9/1		1/0	8/1		1/0
Maintain head/finger position	9/1		1/0	9/1		1/0
Remove finger on hearing device’s signal	2/0		0/0	2/0		0/0
Complete task without level 1 prompt	9/1		1/0	7/1		0/0
Complete task without level 2 prompt	9/1		1/0	7/1		0/0
Complete task without level 3 prompt	9/1		1/0	9/1		0/0
Task 4A	Day 5		Day 33	Day 5		Day 33
Aim device towards object	9/1		2/1	9/1		2/1
Maintain head/finger position	9/1		2/1	9/1		2/1
Remove finger on hearing device’s signal	2/0		0/0	2/0		0/0
Complete task without level 1 prompt	9/1		2/1	9/1		2/1
Complete task without level 2 prompt	9/1		2/1	9/1		2/1
Complete task without level 3 prompt	9/1		2/1	9/1		2/1
Task 5A	Day 5		Day 33	Day 5		Day 33
Aim device towards object	9/1		2/1	9/1		2/1
Maintain head/finger position	9/1		2/1	9/1		2/1
Remove finger on hearing device’s signal	2/0		0/0	2/0		0/0
Complete task without level 1 prompt	9/1		2/1	7/1		2/0
Complete task without level 2 prompt	9/1		2/1	8/1		2/1
Complete task without level 3 prompt	9/1		2/1	9/1		2/1
Task 6A	Day 5		Day 33	Day 5		Day 33
Aim device towards object	9/1		2/1	9/1		2/1
Maintain head/finger position	9/1		2/1	9/1		2/1
Remove finger on hearing device’s signal	5/0		0/0	5/0		0/0
Complete task without level 1 prompt	9/1		2/1	6/0		1/1
Complete task without level 2 prompt	9/1		2/1	8/1		2/1
Complete task without level 3 prompt	9/1		2/1	8/1		2/1
Task 7A	Day 5		Day 33	Day 5		Day 33
Aim device towards object	9/1		1/0	9/1		1/0
Maintain head/finger position	9/1		1/0	9/1		1/0
Remove finger on hearing device’s signal	2/0		0	2/0		0
Complete task without level 1 prompt	9/1		1/0	6/1		1/0
Complete task without level 2 prompt	9/1		1/0	9/1		1/0
Complete task without level 3 prompt	9/1		1/0	9/1		1/0A

A: ^a^Test Items: abbreviated action. Refer to Additional File 1 for full description of item being evaluated. ^b^In four participants with CLN3 disease, feasibility was evaluated as part of Function Test at visit Day 5. Refer to Table B

B: ^a^Test Items: abbreviated action. Prompting levels 1 = verbal reminders, 2 = verbal reminder and specific corrective instructions, and 3 = verbal and non-verbal reminder and specific instructions. Refer to Additional File 2 for full description of item being evaluated

Parental perception of feasibility was evaluated at Day 5. Eight of 10 participants scored 6 or greater on the Feasibility Questionnaire. All (10/10) parents deemed the device’s user instruction simple to follow (Additional File 11). Most parents of individuals with CLN3 disease rated the device easy for their child to use around the house (8/9) and use independently (7/9). Conversely, most (6/9) parents of individuals with CLN3 disease did not find it easy for their child to use the device outside of the house. The parent of the individual without CLN3 disease rated the device as easy to use in or out of the house independently. All parents of children with CLN3 disease (*n* = 9) rated the device as a potentially useful assistive tool. However, not all think their child would use the device (6/9 would use), or would recommend the device to individuals with CLN3 disease (7/9 would recommend).

#### Efficacy

On Day 1, we evaluated participants’ ability to recognize text, color, and faces without using the OrCam MyEye2 (Table 3 level A tasks). When considering any text recognition task, 11–25% (1–2) of the evaluated participants with CLN3 disease completed the task correctly (Table 3). Higher proportion of CLN3 participants correctly completed color (5/9) and face (3/9) recognition tasks. Except for menu item selection, CLN3 participants who correctly completed level A also correctly completed level B tasks combining text or color recognition and information processing (Table 3). The non-CLN3 participant correctly completed the color, face, and two of the five text recognition tasks (Table 3).

**Table 3:** Function test task completion frequency by participants at visits days 1, 5 and 33

Test Items	Number of participants, (completed task/evaluated^a^)					
	CLN3			Non-CLN3		
	Day 1	Day 5	Day 33^b^	Day 1	Day 5	Day 33
Text recognition						
1A. Recognize school-related document^c, e^	0/9	9/9	2/2	0/1	1/1	1/1
2A. Recognize options from a printed menu^c, e^	1/9	9/9	2/2	0/1	1/1	1/1
3A. Recognize a room sign^d^	2/8	9/9	1/1	1/1	1/1	NA
4A. Recognize a printed bin label on a drawer^d, e^	1/9	9/9	2/2	1/1	1/1	1/1
5A. Recognize text on a page in a book^c, e^	0/8	9/9	2/2	0/1	1/1	1/1
Color recognition						
6A. Recognize the color of a uniformed, single, primary colored item^e^	5/8	9/9	2/2	1/1	1/1	1/1
Face recognition						
7A. Recognize a facing person following prompt	3/8	9/9	1/1	1/1	1/1	NA
7B. Recognize a person without prompt	3/3	9/9	1/1	1/1	1/1	NA
Text/Color recognition and Information processing						
1B. Identify the correct subject between two school documents^c, e^	NA	7/9	1/2	0/1	1/1	1/1
2B. Recognize and select food item from a menu^c, e^	0/1	9/9	2/2	0/1	1/1	1/1
3B. Identify which of two labeled doors to enter	2/2	9/9	1/1	0/1	1/1	NA
4B. Recognize and choose one of two drawer label as directed^d, e^	1/1	7/9	2/2	1/1	1/1	1/1
5B. Answer specific relating to content from a page of a book^c, e^	NA	9/9	2/2	0/1	1/1	0/1
6B. Recognize and choose one of two colored items as directed^e^	5/5	8/9	2/2	1/1	1/1	1/1

^a^Evaluated task. If the participant did not complete A-level task, then B-level task would not be evaluated or counted. For logistical purpose and progression of task difficulty, tasks were evaluated in the following order: 4,1,2,5,6,3,7. One CLN3 participant became emotionally upset during Day 1 Function Test (where the test device was not used) after the initial 3 tasks and testing was terminated. The participant was more agreeable to the remainder of the study training and evaluations. ^b^Optional Day 33. One CLN3 participant did optional Day 33 visit in person. One CLN3 and the non-CLN3 participants did optional visit Day 33 remotely. Test items evaluated during a remote visit included 1,2,4,5,6. ^c^Small-font text recognition task. ^d^Large-font text recognition task. ^e^Test items evaluated during optional Day 33 remote visits

**Fig. 4:**
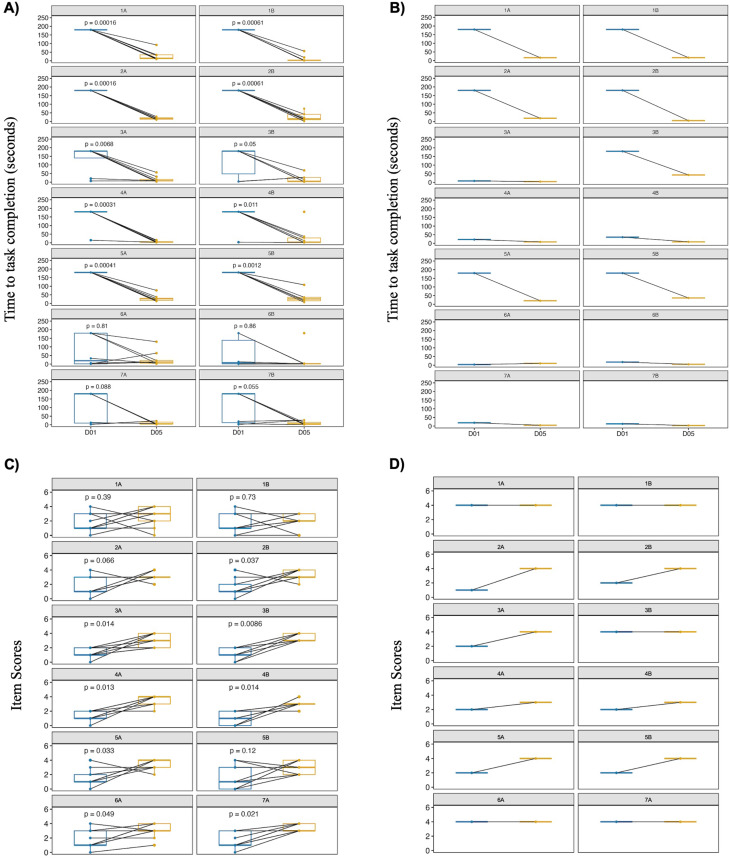
Direct assessments and parental observation of device efficacy. Time taken to complete a task without (visit Day 1) as compared to with (visit Day 5) the device by participants during the Function Test (**A**,** B**). The tasks are indicated in the gray heading of each graph panel and correspond with those in Table 3. Parental assessment of participants’ ability to complete tasks without (visit Day 1) as compared to with (visit Day 5) the test device (**C**,** D**). The tasks are indicated in the gray heading of each graph panel and correspond with those in Table 4. CLN3 participants, *n* = 9 (**A**,** C**). Non-CLN3 participant, *n* = 1 (**B**,** D**). D01 = visit Day 1. D05 = visit Day 5

On Day 5, we evaluated the participants’ use of the OrCam MyEye2 to complete the same Day 1 tasks. For text recognition, all nine participants with CLN3 disease correctly completed all level A text, color, and facial recognition tasks (Table 3). For level B, two CLN3 participants did not correctly complete text-related tasks and one, the color-related task. The non-CLN3 participant completed all level A and B tasks correctly. Time to complete tasks by CLN3 participants notably decreased on Day 5, with those for both small- (items 1, 2, 5) and large- (items 3, 4) font text recognition having (*p* < 0.001) for level A and (*p* < 0.05) for level B tasks (Fig. 4A, Additional File 12). The reductions in time for tasks involving color (item 6) and face (item 7) recognitions were variable for CLN3 participants. The non-CLN3 participant reduced time to complete tasks associated with small-font text recognition (Fig. 4B).

**Table 4:** Parental feedback on device performance

Feedback Category	Number of selections/responses (*n* = 10)
Task performance needing improvement	
More accurate color identification	5
Better recognition of or discrimination between text versus non-text	3
Operational parameters needing more adaptability, flexibility, or sensitivity	
Less dependence on background lighting for recognition of text, colors, or faces	5
Less restriction on area of text captured	6
Less dependence on head positioning or aligning of device to object	3
Better variety of recognized text fonts	3
Better recognition of text boundaries (e.g., column/page breaks, outside of or underneath label)	4
Longer lasting battery	3
Potential additional task capability	
Sensor capability for orientation and mobility (e.g., detecting street obstacles, potential collisions)	3
Recognizing natural speech commands	1

Only 3/10 participants elected to do the optional 1-month (Day 33) visit, with two opting for a remote evaluation. Both CLN3 participants correctly completed all tasks, except for one small-font text identification level B task (Table 3). The non-CLN3 participant opted for a remote Day 33 evaluation and correctly completed all tasks, except for one small-font text recognition level B task.

### Ability survey and device use dairy

Of the tasks matching to the capabilities of the device, parents rated improvements in the CLN3 participants’ ability to recognize small- and large-font text and faces at visit Day 5 versus 1 (Fig. 4C). Parents noted no significant improvement with tasks involving recognition of text on school-related documents or colors. For the non-CLN3 participant, Day 5 ability score increased for tasks involving mainly small-font text recognition (Fig. 4D).

Tasks on which the OrCam MyEye2 did not perform to parental expectation included color recognition and text versus non-text discrimination (Table 4). Operational parameters that contributed to suboptimal use of the device in general, and more specifically for the studied cohort of children with blindness and neurocognitive challenges, included requirements for brightly lit background and accurate and steady head positioning. Parents also suggested improvements in the area of recognition of text font varieties and text boundaries. Several parents indicated that adding a capability of the device to help with orientation and mobility (e.g., detection of curbs or objects) and recognition of natural speech commands would be desirable.

## Discussion

Visual impairment in children with developmental delay or individuals with intellectual/multiple disabilities is present at a high frequency [25–27]. Further research to identify additional resources and optimized approaches for accommodation is critical as the multiplicity of disabilities likely complicates the general application of typical adaptive methods and limits the timing for interventions. These aspects are apparent in the pediatric blindness and neurodegenerative condition of CLN3 disease. The combination of progressive decline in neurocognition, vision, speech, and motor in CLN3 disease engenders a complex presentation for which to develop low-vision accommodations and management. In this study, surveys of participants and parents/caregivers quantify the significant effect of low vision on quality of life and underscore their desire that vision be a top area for further CLN3 disease research. Caregivers for individuals with CLN3 disease reported much lower scores on the LVQoL than the comparable data on adults with normal vision (who scored at 64–97% of maximum possible score) [22]. 

The OrCam MyEye 2 was advertised to have multiple performance functions and simple operational procedures. From our pilot study of nine children with CLN3 disease and one with non-CLN3 disease related low vision, use of the device is feasible, without incurring any major adverse event. Efficacy evaluations based on study- and device-specific designed tasks and questionnaires showed improved performance during a 5-day use of the device by children with CLN3 disease.

Current research into treatment approaches for the vision loss associated with CLN3 disease is challenging given the rarity of the condition and lack of animal models that recapitulate human retinal presentation to evaluate the efficacy of candidate therapies. Treatment trials in development (NCT03770572, NCT05174039, NCT04637282) do not specifically target the eyes. Transduction of patient-derived retinal neurons with an AAV2-CLN3 vector or subretinal injections of the vector into wild-type mice led to expression of appropriate products without toxicity [28]. In Cln3 mouse models, AAV vector-mediated gene therapy approaches showed disparate effects on retinal cell loss [29]. The long-term benefits of these therapeutic interventions remain to be investigated in future clinical studies. Thus, improving accommodative options remains important.

Standard vision accommodative approaches such as enlarged fonts, Braille, and sign language are less feasible and enduring in individuals with CLN3 disease, presumably due to the progressive loss of both vision and neurocognitive abilities. Advances in technology such as optical character recognition, text-to-speech synthesizers and voice over programs have allowed for increased accommodation and application for individuals with CLN3 disease in the early years of the disease [11, 30]. As neurocognitive, speech, and motor functions decline, the technologies lose applicability due to innate design requiring intact cognitive and motor coordinations (e.g., Braille, sign language), or insufficient capability to parse varying degrees of speech clarities (e.g., speech recognition devices). Expert recommendations for rehabilitative program and resources for children with CLN3 disease encompass multiple disciplines and emphasize built-in flexibility and timely adaptability to address changes in the child’s performance [17, 30, 31]. 

This study has several important limitations, some of which may be informative for the design of future trials. The study terminated early because of slow enrollment. A contributing factor to this was difficulty recruiting similar-age children without CLN3 disease and with comparable severe vision loss and typical neurodevelopment. Although anecdotal reports suggest feasibility of OrCam MyEye 2 use in children [19, 20], the scarcity of referred children who had comparable level of vision loss and typical neurodevelopment precluded rigorous assessments of whether limitations in device feasibility were due to age (i.e., pediatric cohort), neurodevelopmental level, or the multisystemic involvement of CLN3 disease. The data evaluated are from short-term use of the device, as only 3 of the 10 participants chose to continue with the optional 33-day study despite a possibility for remote evaluation. Per study design, use of the device was limited to standardized testing environments or in constant parental oversight. The results from this study suggest that a follow up study focusing on a longer use duration (e.g., 30 days) and incorporating use of the device in other settings (e.g., home, school, social events) is needed to evaluate further the applicability of the device in the CLN3 population. It is notable that current individual accessibility to the device is limited due to cost. Thus, the most likely option for affected children to gain access to the device, as well as for the outlined follow up study to be conducted, would involve school or community low-vision therapy and rehabilitation programs.

## Conclusions

The results from this pilot study suggest the OrCam MyEye 2 as a potential additional safe augmentative communication device option for children with CLN3 disease. The simple operating maneuvers that are more motor and positionally based (i.e., keeping head still and facing straight ahead) may be an applicable option for training children with multiple disabilities to result in task performance improvement as seen in this study. From our experience during the study and based on parental feedback, use of the device by children with CLN3 disease requires assistance and reminders from caregiver. Longer intervention may allow determination of whether this would lead to greater independence in task performance. Several areas for device design modifications are provided that may improve the daily and long-term applicability of this device in children with CLN3 disease. Overall, more intensive evaluation of the device and how training can be used to generalize its use both in and out of the home is warranted to assess its full applicability for children with CLN3 disease, and for children with other multiple disability conditions.

## Electronic Supplementary Material

Below is the link to the electronic supplementary material.

Supplementary Material 1: **Additional File 1**. Feasibility test standard operating procedure.

Supplementary Material 2: **Additional File 2**. Function assessment standard operating procedure.

Supplementary Material 3: **Additional File 3**. OrCam training manual.

Supplementary Material 4: **Additional File 6**. Applicability questionnaire.

Supplementary Material 5: **Additional File 7**. Ability questionnaire.

Supplementary Material 6: **Additional File 8**. Feasibility questionnaire.

Supplementary Material 7: **Additional File 4**. Optional Day 33 study extension. **Additional File 5**. OrCam MyEye 2. **Additional File 9**. Parental choice of signs or symptoms of CLN3 disease they would like to see being addressed by future interventions. **Additional File 10**. PedEyeQ scaled scores of parental perspectives at baseline visit. **Additional File 11**. Evaluation of feasibility of using the device by parental questionnaire at visit Day 5. **Additional File 12**. Time-to-task completion of Function Test items.

## Data Availability

All data generated or analyzed during this study are included in this published article and its supplementary information files.
