# Supplementary material for: Prospective pilot safety, feasibility study of an optic-to-audio device for children with CLN3 disease
Source: Orphanet J Rare Dis. 2026 Apr 3;21:199. doi: 10.1186/s13023-026-04319-0 (PMC13173725; doi:10.1186/s13023-026-04319-0)
Supplement: Supplementary file 1 — Supplementary Material 1: Additional File 1. Feasibility test standard operating procedure. [file 13023_2026_4319_MOESM1_ESM.pdf]

ADDITIONAL FILE 1

Protocol: Pilot Study of an Optic-to-Audio Device in a Pediatric Cohort with CLN3-related conditions or Low Vision

iRIS Number: 000414

Form: Feasibility Test SOP

Version: October 1, 2021

References: Moisseiev E and Mannis MJ. 2016. JAMA Ophthalmology 134(7):748-52.

Test Overview

The Feasibility Test will be used to evaluate the feasibility of using OrCam MyEye 2 by the participants.

Test Administration

The Feasibility Test will be evaluated using the below methods. Methods 1 will be completed by study team members during training session and Function test. Methods 2 and 3 will be completed by parents/guardians using paper or electronic formats.

1) Feasibility Test: an interactive test to evaluate ability to perform basic actions involved in using the device. It is scored as 0=No, 1=Yes.
2) Feasibility Questionnaire: an item questionnaire using a Likert rating scale
3) Device Use Diary: a log of daily use (duration, activity type)

Testing Administrator: a trained team member who will be working directly with the study participant during the test.

Scoring Administrator: a trained team member who will be scoring, keeping time, and recording these on CRFs during the test.

*****If participant scores 0 on any of Items #1-6, exploratory items in Feasibility and Function Tests will not be done.*****

FEASIBILITY TEST

Test Overview

The Feasibility Test contains items to evaluate the feasibility of using the device, in this case the OrCam MyEye 2. The Feasibility Test should be administered and scored by trained evaluators only. To standardize administration and scoring, parents should be asked to refrain from giving verbal cues or physical assistance to the child in using the OrCam to complete the Feasibility Test items. The children will be informed that their parents are observing only and will not be helping during the testing.

Feasibility Test shall be done at the following visits: baseline (with the device in Off mode), at 1-week (with the device in Off or On mode), and 1-month evaluations (with the device in Off or On mode). Feasibility Test can be scored as part of Function Test and not be administered separately during the 1-week and 1-month evaluations.

Items 1-6 constitute the main score. Items 7-9 are exploratory.

Additional Materials needed for test administration are listed in the Table below.

Item #	Activity	Scoring	Additional Materials Needed
**1**	Participant able to maintain wearing eyeglass with mounted device in place for duration of the Feasibility or Function test.	0=No; 1=Yes	OrCam MyEye 2 and accessories
**2**	Participant able to maintain head looking straight ahead for 10 seconds	0=No; 1=Yes	None
**3**	Participant able to position pointer finger in front of face/head	0=No; 1=Yes	None
**4**	Participant able to maintain positioned pointer finger with nailbed towards face for 10 seconds	0=No; 1=Yes	None
**5**	Participant able to raise hand, palm outward, in front of face and hold for 10 seconds (stop gesture for OrCam)	0=No; 1=Yes	None
**6**	Participant able to tap once towards the front half of the device	0=No; 1=Yes	OrCam MyEye 2 and accessories
**7**	Participant able to raise arm in posture of checking wristwatch	0=No; 1=Yes	None
**8**	Participant able to maintain arm in position as stated in item 7 for 10 seconds	0=No; 1=Yes	None
**9**	Participant able to mount device to magnetic attachment on eyeglass in correct orientation	0=No; 1=Yes	OrCam MyEye 2 and accessories

Test Administration

Testing Set Up

Quiet, well-lit room that is exposed to minimal traffic and interruptions.
People in the room shall include study participants, the Testing Administrator and secondary Testing Administrators. Parents/guardians may be in the room but will be asked to refrain from providing instruction or assistance during the testing.

Participant will be seated on a chair at a table, facing the Testing Administrator. A screen device (desktop, laptop, or iPad) with internet access shall be available.

The following items will be needed:

- Charged OrCam MyEye 2
- Eyeglass with mounting attachment
- Testing Administration Instructions/SOP
- Case Report/Scoring Form
- Stop watch
- Portable lamp/flashlight for additional illumination

Orientation

Testing Administrator verbalizes:

"Throughout this week, you will learn to use a device that may help with some of the activities that you do every day, such as reading, identifying colors, and recognizing faces. The device is called the OrCam MyEye, or OrCam for short. Have you ever worn eyeglasses? Ok, you will wear a pair of eyeglasses. Then, the OrCam will stick to the side of the eyeglasses near your ear.

The OrCam has a camera, and a speaker that will talk into your ear. I will teach you how to use the device for the activities. Your parent and other people will watch and take notes. They will stay quiet during our activities. Before we start, do you have any question? Okay, let's begin."

Introduction to the Device

Testing administrator places the eyeglasses in front of participant and verbalizes:

"I just put a pair of eyeglasses in front of you. Go ahead and feel them. On one side of the eyeglasses, you will feel a bump. You want to put the bump to your right-hand side. That is where the OrCam will attach to the eyeglasses."

After 10 seconds, Testing administrator places the OrCam device in front of participant and verbalizes:

"Please put the eyeglasses down. I just put the OrCam in front of you. Go ahead and feel it."

After 10 seconds, Testing administrator verbalizes:

"Please put the OrCam down. Now, I will show you how to attach the OrCam to the eyeglasses. To do this, I will touch and move your hands. Let me know if any of this makes you uncomfortable."

Testing administrator positions the OrCam with the metal end pointing towards and the ridged line facing to the right of the participant.

Testing administrator verbalizes and correspondingly moves the participant's hand:

"I will move your hand to feel the pointy end of the OrCam. You want to put the pointy end towards you. Now, I will move your hand to feel the raised line on the side of the OrCam. You want to put the raised line on your right-hand side.
Now let's bring the OrCam close to the eyeglasses. The OrCam will attach to the bump on the eyeglasses like that."

These processes can be repeated during device training. Thus, if participant appears to struggle with mounting the device, Testing Administrator can facilitate this so that testing can proceed to the Feasibility items.

If participant appears fatigued or inattentive, Testing Administrator may announce a break for a few minutes before starting the Feasibility Test.

Directions to Study Participants

Testing administrator verbalizes:

"Now I am going to ask you to do a few different activities. These are things that you would normally do. I will not ask you to do anything scary or hurtful. I will ask you to do these activities without help from your parents. Some of these you may or may not be able to do. That's ok! I only ask that you try your best.

For each activity I will first explain what I will ask you to do. Then, I will ask you to do the activity a few times to see how <u>you</u> do it. You may hear me move around the table as I help you."

Testing Administration

During the practice run, the Testing Administrator may provide verbal instructions and physical assistance to the participant to demonstrate the correct performance.

During the actual scored test (i.e., when the participant is asked to do the activity "one more time"), the Testing administrator may only provide the verbal instructions.

Item #1 Participant able to maintain wearing eyeglass with mounted device in place for duration of the Feasibility or Function test. (At baseline evaluation, the device will be in Off mode.)

Testing Administrator signals test item number and establishes eye contact with Scoring Administrator. Testing Administrator verbalizes:

"Please put on the eyeglasses with the OrCam on it. Keep them on until we are done with the activities."

Testing Administrator signals the score to Scoring Administrator for confirmation.

0=no, participant cannot keep eyeglass on during the test.

1=yes, participant can keep eyeglass on during the test.

Item #2 Participant able to maintain head looking straight ahead for 10 seconds.

Testing Administrator signals test item number and establishes eye contact with Scoring Adminstrator. Testing Administrator verbalizes:

"For this activity, I am going to ask you to keep your head straight and still up to the count of 10."

Testing Administrator counts out loud (one one thousand, two one thousand, etc.) to 10. After the count of 10, verbalizes:

"You may relax."

If participant can do the task, may skip practice #2. If participant cannot do it, provide corrective instructions (verbal and physical assistance), and repeat the practice one more time: "This is another try. Please keep your head straight and still up to the count of 10."

Testing Administrator signals to the Scoring Administrator a scored test run by starting the instruction with "Let's do this one more time." Testing Administrator verbalizes:

"Let's do this one more time. Please keep your head straight and still up to the count of 10."

Testing Administrator counts out loud to 10. **This will be the scored test run.**

After the count of 10, Testing Administrator verbalizes:
“You may relax.”

Testing Administrator signals the score to Scoring Administrator for confirmation:
0=no, participant cannot keep head straight and still for 10 seconds (up to the count of 10) on the test run.
1=yes, participant can keep head straight and still for 10 seconds (up to the count of 10) on the test run.

Item #3   Participant able to position pointer finger in front of face/head, with the nail towards the face/head.

Testing Administrator signals test item number and establishes eye contact with Scoring Adminstrator. Testing Administrator verbalizes:
“For the next activity, I am going to ask you to put your pointing finger out in front of your face like a number one. I will move your finger to show you what I mean.”

Testing Administrator adjusts the participants’ finger so that the nailbed is facing towards the participant, and the finger is raised to the level of the participant’s nose. Testing Administrator verbalizes:
“When I ask you to put your pointing finger out in front of your face, you do it like that. You may relax. Now, please put your pointing finger out in front of your face like a number one, and keep it raised as high as your nose.”

If participant can do the task, may skip practice #2. If participant is cannot do it, provide corrective instructions (verbal and physical assistance), and repeat the practice one more time: “This is another try. Please put your pointing finger out in front of your face, and keep it raised as high as your nose.”

Testing Administrator signals to Scoring Administrator a scored test run by starting the instruction with “Let’s do this one more time.” Testing Administrator verbalizes:
“Let’s do this one more time. Please put your pointing finger out in front of your face, and keep it raised as high as your nose.”
This will be the scored test run.

Testing Administrator verbalizes:
“You may relax.”

Testing Administrator signals the score to Scoring Administrator for confirmation:
0=no, participant cannot position pointer finger in front of face with nail towards the face on the test run.
1=yes, participant can position pointer finger in front of face with nail towards the face on the test run.

Item #4 Participant able to position pointer finger in front of face/head, with the nail towards the face/head, and hold still for 10 seconds.

Testing Administrator signals test item number and establishes eye contact with Scoring Administrator. Testing Administrator verbalizes:

"For the next activity, you are going to repeat exactly what you just did and hold still in that position while I count to 10. "

Testing Administrator counts out loud up to 10. If participant can do the task, may skip practice #2. If participant is cannot do it, provide corrective instructions (verbal and physical assistance), and repeat the practice one more time: "This is another try. Please put your pointing finger out in front of your face, keep it raised as high as your nose, and hold still while I count to 10."

Testing Administrator signals to Scoring Administrator a scored test run by starting the instruction with "Let's do this one more time." Testing Administrator verbalizes:

"Let's do this one more time. Please put your pointing finger out in front of your face, keep it raised as high as your nose, and hold still while I count to 10."

Testing Administrator counts out loud up to 10. **This will be the scored test run.**

After the count of 10, Testing Administrator verbalizes:

"You may relax."

Testing Administrator signals the score to Scoring Administrator for confirmation:

0=no, participant cannot position pointer finger in front of face with nail towards the face and hold still for 10 seconds on the test run.

1=yes, participant can position pointer finger in front of face with nail towards the face and hold still for 10 seconds on the test run.

Item #5 Participant able to raise hand, palm outward, in front of face and hold for 10 seconds (stop gesture for OrCam)

Testing Administrator signals test item number and establishes eye contact with Scoring Adminstrator. Testing Administrator verbalizes:

"For the next activity, I am going to ask you to put your hand in front of your face, like a stop sign or a high five . Keep your hand raised as high as your nose. I will move your hand to show you what I mean."

Testing Administrator adjusts the participants' hand so that the nailbeds are facing towards the participant, and the hand is raised to the level of the participant's nose. Testing Administrator verbalizes:

"When I ask you to put your hand out in front of your face, you do it like that. This time you will also need to keep your hand raised while I count to 10. You may relax. Now, please put out your hand like a stop sign or high five, keep it raised as high as your nose, and hold still while I count to 10."

Testing Administrator counts out loud up to 10. If participant can do the task, may skip practice #2. If participant cannot do it, provide corrective instructions (verbal and physical assistance), and repeat the practice one more time: "This is another try. Please put your hand in front of your face, keep it raised as high as your nose, and hold still while I count to 10."

The Testing Administrator signals to the score keeper a scored test run by starting the instruction with "Let's do this one more time." Testing Administrator verbalizes:

"Let's do this one more time. Please put your hand in front of your face, keep it raised as high as your nose, and hold still while I count to 10."

Testing Administrator counts out loud up to 10. **This will be the scored test run.**

After the count of 10, Testing Administrator verbalizes:

"You may relax."

Testing Administrator signals the score to Scoring Administrator for confirmation:

0=no, participant cannot put hand in front of your face in stop gesture or cannot hold still for 10 seconds on the test run.

1=yes, participant can put hand in front of your face in stop gesture and hold still for 10 seconds on the test run.

Item #6 Participant able to tap once at the front half of the device.

Testing Administrator signals test item number and establishes eye contact with Scoring Administrator. Testing Administrator verbalizes:

"For the next activity, I am going to ask you to tap the OrCam once on the raised line, using your pointing finger. I will move your finger to show you what I mean. Start at the front and slide your finger back to find the raised line."

Testing Administrator guides the participants' finger to the front of the OrCam and demonstrates the tapping on raised line motion. Testing Administrator verbalizes:

"When I ask you to tap the OrCam once on the raised line, you do it like that and keep your head still up to the count of 3. You may relax. Now please tap the OrCam **once** on the raised line and keep your head still up to the count of 3."

Testing Administrator counts out loud up to 3. If participant can do the task, may skip practice #2. If participant cannot do it, provide corrective instructions (verbal and physical assistance), and repeat the practice one more time: "This is another try. Please tap the OrCam **once** on the raised line and keep your head still and relax your hand."

The Testing Administrator signals to the score keeper a scored test run by starting the instruction with "Let's do this one more time." Testing Administrator verbalizes:

"Let's do this one more time. Please tap the OrCam **once** towards the front and keep your head still while I count to 10."
This will be the scored test run.

Testing Administrator signals the score to Scoring Administrator for confirmation:
0=no, participant cannot tap the OrCam once towards the front or cannot hold head still for 10 seconds on the test run.
1=yes, participant can tap the OrCam once towards the front and hold head still for 10 seconds on the test run.

If **proceeding** with exploratory items, Testing Administrator verbalizes:
"You may relax."
If **not proceeding** with exploratory items (e.g., participant is fatigued or inattentive, or if time is running out), Testing Administrator verbalizes:
"This is the end of these activities. Thank you for working with me. You may relax while we get set up for other activities."

Item #7   Participant able to raise arm in posture of checking wristwatch.

Testing Administrator signals test item number and establishes eye contact with Scoring Adminstrator. Testing Administrator verbalizes:
"For the next activity, I am going to ask you to raise your arm like you are looking at the back of your wrist. Keep your arm raised as high as your nose. I will help move your arm to show you what I mean."

Testing Administrator guides the participants' arm to line up the back of the wrist to the OrCam. Testing Administrator verbalizes:
"When I ask you to raise your arm like you are looking at the back of your wrist, you do it like that. You may relax. Now, please raise your arm like you are looking at the back of your wrist, and keep your arm raised as high as your nose."

If participant can do the task, may skip practice #2. If participant cannot do it, provide corrective instructions (verbal and physical assistance), and repeat the practice one more time: "This is another try. Please raise your arm like you are looking at the back of your wrist, and keep your arm raised as high as your nose."

The Testing Administrator signals to the score keeper a scored test run by starting the instruction with "Let's do this one more time." Testing Administrator verbalizes:
"Let's do this one more time. Please raise your arm like you are looking at the back of your wrist and keep your arm raised as high as your nose."
This will be the scored test run.

Testing Administrator verbalizes:
"You may relax."

Testing Administrator signals the score to Scoring Administrator for confirmation:
0=no, participant cannot raise arm like checking wristwatch on the test run.
1=yes, participant can raise arm like checking wristwatch on the test run.

Item #8 Participant able to maintain arm in position in item #7 for 10 seconds.

Testing Administrator signals test item number and establishes eye contact with Scoring Adminstrator. Testing Administrator verbalizes:
"For the next activity, you are going to repeat exactly what you just did and hold still in that position while I count to 10."

Testing Administrator counts out loud up to 10. If participant can do the task, may skip practice #2. If participant cannot do it, provide corrective instructions (verbal and physical assistance), and repeat the practice one more time: "This is another try. Please raise your arm like you are looking at the back of your wrist. Keep your arm raised as high as your nose while I count to 10."

The Testing Administrator signals to the score keeper a scored test run by starting the instruction with "Let's do this one more time." Testing Administrator verbalizes:
"Let's do this one more time. Please raise your arm like you are looking at the back of your wrist. Keep your arm raised as high as your nose while I count to 10."
Testing Administrator counts out loud up to 10. **This will be the scored test run.**

After the count of 10, Testing Administrator verbalizes:
"You may relax."

Testing Administrator signals the score to Scoring Administrator for confirmation:
0=no, participant cannot raise arm like checking wristwatch and keep it raised for 10 seconds on the test run.
1=yes, participant can raise arm like checking wristwatch and keep it raised for 10 seconds on the test run.

Item #9 Participant able to mount device to magnetic attachment on eyeglass in correct orientation

Testing Administrator signals test item number and establishes eye contact with Scoring Adminstrator. Testing Administrator verbalizes:
"For the next activity, I am going to ask you to attach the camera to the eyeglasses, like you did at the beginning."

Testing Administrator positions the OrCam with the metal end pointing towards and the ridged line facing to the right of the participant. Testing Administrator verbalizes and correspondingly moves the participant's hand:

"I will move your hand to feel the pointy end of the OrCam. You want to put the pointy end towards you. Now I will move your hand to feel the raised line on the side of the OrCam. You want to put the raised line on your right-hand side. Now let's bring the OrCam close to the eyeglasses. The OrCam will attach to the bump on the eyeglasses like this. You may relax, and then we will try again. Now, please attach the camera to the eyeglasses."

Allot 10 seconds for the action to be completed. If participant can do the task, may skip practice #2. If participant cannot do it, provide corrective instructions (verbal and physical assistance), and repeat the practice one more time: "This is another try. Please attach the camera to the eyeglasses."

The Testing Administrator signals to the score keeper a scored test run by starting the instruction with "Let's do this one more time." Testing Administrator verbalizes:

"Let's do this one more time. Please attach the camera to the eyeglasses."

This will be the scored test run.

Testing Administrator verbalizes:

"This is the end of these activities. Thank you for working with me. You may relax while we get set up for other activities."

Testing Administrator signals the score to Scoring Administrator for confirmation:

0=no, participant cannot attach the camera to the eyeglasses.
1=yes, participant can attach the camera to the eyeglasses.
