# Supplementary material for: Prospective pilot safety, feasibility study of an optic-to-audio device for children with CLN3 disease
Source: Orphanet J Rare Dis. 2026 Apr 3;21:199. doi: 10.1186/s13023-026-04319-0 (PMC13173725; doi:10.1186/s13023-026-04319-0)
Supplement: Supplementary file 2 — Supplementary Material 2: Additional File 2. Function assessment standard operating procedure. [file 13023_2026_4319_MOESM2_ESM.pdf]

ADDITIONAL FILE 2

Protocol: Pilot Study of an Optic-to-Audio Device in a Pediatric Cohort with CLN3-related conditions or Low Vision

iRIS Number: 000414

Form: Function Assessment SOP

Version: October 12, 2021

References: Moisseiev E and Mannis MJ. 2016. JAMA Ophthalmology 134(7):748-52.

OrCam product information and User Guide

<u>**Table of Contents**</u>

I. **Test Overview**

II. **Test Administration**

A. *Testing Set Up*
B. *Testing Materials*
C. *Testing Administration*
1. <u>Administration of Prompts</u>
2. <u>Scoring</u>
a. Likert Scoring
b. Timing

III. **Directions to Study Participants**
A. *Baseline*
B. *1 Week/1 Month*

Test Overview

The Function Test contains items to evaluate the feasibility and efficacy of accomplishing the main or relevant advertised capabilities of the OrCam MyEye 2 device:
1. manual recognition of standard printed text and translation of the text to audio inputs
2. manual recognition of faces and association to name programmed into the device
3. manual identification of color and translation to audio inputs

The Function Test should be administered by a trained evaluator only. To standardize administration and scoring, parents should be asked to refrain from assisting the child in using the OrCam to complete the Function Test items. Function Test shall be done at the following visits: baseline (without using the device), at 1-week (using the device), and 1-month evaluations (using the device).

The test items on the Function Test are evaluated and scored at two levels:
a) involves the participant demonstrating correct use of the OrCam device. This assesses feasibility.
b) involves the participant demonstrating use of the OrCam device to accomplish the required task. This assesses device efficacy.
c) involves the participant demonstrating use of the OrCam device in a functional manner in a way that is applicable to their daily life. This assesses device efficacy.

Items 1-7 constitute the main score. **Items 8-10 are exploratory**.
Items 1,2,4,5,6 can be administered both in-person and remotely.

Additional Materials needed for test administration are listed in the table below.

#	Level	Item	Additional Materials Needed
1	A	Using OrCam to recognize a school-related document (test admin hands participant document and ask participant to use OrCam and tell admin what is on the document)	School-related documents, clipboard, book stand
	B	Using OrCam to identify the correct subject (asked by Administrator 1) of two school-related documents presented (test admin places two documents in front of participant and ask participant to use OrCam to find one on subject X and hand to admin)	School-related documents, clipboard, book stand
2	A	Using OrCam to recognize options from a printed Menu. (test admin hands participant Menu, asks participant to use OrCam and tell admin any item listed on Menu)	Menu, clipboard, book stand
	B	Using OrCam to select food item (test admin asks participant to use OrCam, choose, and verbalize a preferred Menu item)	Menu, clipboard, book stand
3	A	Using OrCam to recognize a room sign (test admin takes participant outside of testing room, positions participant in front of room sign and asks participant to use OrCam and tell admin the room's label)	Room sign
	B	Using OrCam to identify which of two doors participant would enter (based on their need/preference).	Room signs
4	A	Using OrCam to recognize a printed label on a drawer (shirts, pants, socks).	container with a different label (shirts, pants, or socks) on each drawer
	B	Using OrCam to recognize one of two drawer labels and open the appropriate drawer as directed by the examiner.	container with a different label (shirts, pants, or socks) on each drawer
5	A	Using OrCam to recognize text on a page in a book (test admin opens a book to a page, hands to participant, asks participant to use OrCam and tell admin any word from that page)	children's books, book stand
	B	Using OrCam to answer specific question relating to content from a page of a book (test admin opens book to a page, hands to participant, asks participant to use OrCam and tell admin X from that page)	children's books, book stand
6	A	Using OrCam to recognize the color of a uniformed, single, primary colored item	colored papers

	B	Using OrCam to recognize one of two colored items and hand over the appropriately colored item as directed by the test admin	colored papers
7	A	Using OrCam recognize a facing person following prompt from the test admin (test admin tells participant: someone will come and sit in front of you. I will ask you to use the OrCam and tell me the name of that person.).	study team member
	B	Using OrCam to recognize a facing person without prompt. (test admin tells participant: someone will come and sit or stand in front of you. You will not hear any word. Please use the OrCam and tell me who is the person in front of you.)	study team member or other providers
8	A	Using OrCam to report the day and date	None
	B	Using OrCam to identify the day and answer test admin's question (how many days until Sunday)	None
9	A	Using OrCam to recognize one of two snack products by its label (e.g., bag of Goldfish Crackers, bag of Teddy Grahams, and bag of pretzels).	snack-sized bags, e.g., goldfish cracker, Teddy Grahams, pretzels
	B	Using OrCam to recognize and take the preferred snack option.	snack-sized bags, e.g., goldfish cracker, Teddy Grahams, pretzels
10	A	Using OrCam to recognize options from a menu on a screen device (test admin provides participant with a menu on a screen device, asks participant to tell admin any option from the menu)	screen device (e.g., laptop, iPad), menu list of songs, books, games, etc.
	B	Using OrCam to recognize option from a menu and decide whether an action can be done (test admin provides participant with a menu, asks participant to use OrCam, asks participant whether *an option not included* is there)	screen device (e.g., laptop, iPad), menu list of songs, books, games, etc.

Test Administration

Testing Set Up

Quiet, well-lit room that is exposed to minimal traffic and interruptions.
People in the room shall include study participants, the Administrator 1, and Administrator 2.
Parents/guardians optimally should not be in the room, or if in the room will be asked to refrain from providing instruction or assistance during the testing.

Participant will be seated on a chair at a table, facing the Administrator 1. A screen device with internet access shall be available.

Testing Materials

OrCam MyEye 2 device and ancillary materials
Testing supplemental materials as listed in the table
Testing Administration Instructions/SOP
Case Report/Scoring Form
Stop watch
Portable lamp/flashlight for additional illumination

Test Administration

<u>Administrator 1</u>: a trained team member who will be working directly with the study participant during the test. Is also responsible for scoring participant's performance of test items, and recording of the scores on the CRFs.
<u>Administrator 2</u>: a trained team member who will be keeping lap and total times, recording the total time on CRFs. Is also responsible for setting up materials needed for each test items (e.g., bins, book stands, colored items, etc.)

<u>Administration of Prompts</u>

At baseline, the prompts will consist of re-verbalizing the instruction for the test item.

At 1-week/1-month, Testing Administrator can give each of the below prompt 1x only per test item.

1. Level 1 Prompt: verbal reminder to use the device: "Use the OrCam on your eyeglasses like you did in practice."
2. Level 2 Prompt: verbal reminders to use the device and verbal instructions on how to use the device, e.g.:
   "Feel where the item is."
   "Keep your head straight at the item."
   "Keep your chin up."
   "Keep your head still until after you hear the camera sound."
   "Tap only once on the OrCam."
   "Tap on the raised line."
   "Point your finger at the item with your nail towards your face."
   "Keep your finger still until after you hear the camera sound."
3. Level 3: verbal and non-verbal reminder to use the device, e.g., adjusting the participant's head/finger to aim/point at the object in question

Testing Administrators should always follow a consistent hierarchy of prompts with the study participant.

1. Level 1 prompt should be a verbal reminder to use device.
2. Level 2 prompt should be verbal instructions for how to use device and should be given ~15 seconds after the previous level prompt fails to aid the study participant in completing the task.

3. Level 3 prompt should be physical assistance with using the device and should be given ~15 seconds after the previous level prompt fails to aid the study participant in completing the task. **Note: Testing Administrators should only use a level 3 prompt after level 1 and 2 prompts were administered and were unsuccessful.

<u>Scoring</u>

This will be done by the Administrator 1 in the room, in confirmation with the Administrator 2.
For each item, score:

A. Feasibility of device use, to be done at 1-week and 1-month evaluations

	Evaluations	Score
1	Participant able to aim device towards object	0=No; 1=Yes
2	Participant able to maintain head/finger position until device provided audio inputs (or 5-10 seconds if device malfunctions)	0=No; 1=Yes
3	Participant able to remove finger from object upon hearing device's double beeps/camera sound	0=No; 1=Yes
4	Participant needed level 1 prompt to complete task	0=Yes; 1=No
5	Participant needed level 2 prompt to complete task	0=Yes; 1=No
6	Participant needed level 3 prompt to complete task	0=Yes; 1=No

B. Efficacy of task completion

**Evaluation**	**Score**
Part a) of task completed	0=No; 1=Yes
Part b) of task completed	0=No; 1=Yes
Duration needed to complete part a) task	time (in minutes)
Duration needed to complete part b) task	time (in minutes)

C. Timing (in minutes*)*
Timing should begin after the examiner finishes providing the verbal instruction for each item. Timing should not be stopped until task is completed or discontinued (if prompts are given, timing should continue and should not be stopped and restarted).

Directions to Study Participants

BASELINE FUNCTION TEST

Administrator 1 verbalizes:

"Now, I want to observe how you usually do some activities on your own, such as reading, identifying colors, and recognizing faces. You may be able to do some or none of these activities. For this part it is ok to say you cannot do the activity. Please try your best. If you would like to take a break at any point, please ask. Let's start."

Item #4a Recognize a printed label on a bin/drawer.

Administrator 1 signals test item number/letter and establishes eye contact with Administrator 2.

Administrator 1 places a labeled bin in front of participant. The bin is labeled with single-word items (e.g., "shirts", "pants", "socks") in printed, capital letters, 1-inch in height (Helvetica font, size 72), using black-ink on standard white paper.
Administrator 1 verbalizes:

"For this activity, I am going to ask you about labels. I just put a labeled bin in front of you. I will put your hand where the label is."

Administrator 1 guides participant's hand to the label.

"Please read and tell me what the label says."

Administrator 2 starts stop-watch at the end of verbal instruction for 15 seconds.

Administrator 2 starts stop-watch at the end of verbal instruction for scoring purpose (i.e., only stop when activity has been completed or ended).

If participant declares being unable to do, or has not attempted/completed the task,
Give Level 1 prompt: "[Participant's name], please read and tell me what the label says."
Administrator 2 starts stop-watch at the end of verbal instruction for 15 seconds.

If participant declares being unable to do, then score Items #4a and b as not completed and the duration as the maximum 180 seconds, and move to Item #1.

If participant appears to be trying, then Administrator 1 follows the below prompts.

After 15 seconds,
If participant has not attempted/completed the task, give Level 2 prompt: "[Participant's name], please read and tell me what the label says."
Administrator 2 starts stop-watch at the end of verbal instruction for 15 seconds.

After 15 seconds,
If participant has not attempted/completed the task, give Level 3 prompt: "[Participant's name], please read and tell me what the label says."

Administrator 2 starts stop-watch at the end of verbal instruction for 15 seconds.

After 15 seconds,
If participant has not completed the task, then score Items #4a and b as not completed and the duration as the maximum 180 seconds, and move to Item #1. If participant appears still to be attempting to complete the task, Administrator 1 may elect to help the participant to complete the task though completion will not be scored.

Administrator 1 signals the score to Administrator 2 for confirmation.

Item #4b Recognize one of two bin/drawer labels and select the appropriate bin/drawer as directed by the examiner.

Administrator 1 signals test item number/letter and establishes eye contact with Administrator 2.

Administrator 1 verbalizes:
"For the next activity, I am going to give you 2 labeled bins and ask you to tell me which one says XXXXXX."
Administrator 1 places labeled bin #1 in front of participant.
"I just put bin #1 in front of you. I will put your hand where the label is."
Administrator 1 guides participant's hand to the label.
"Please read to yourself what the label says."
Administrator 1 counts by hand/silently (one one thousand, two one thousand, etc.) to 10.

After 10 seconds, Administrator 1 places labeled bin #2 in front of participant.
Administrator 1 verbalizes:
"Now, I just put bin #2 in front of you. I will put your hand where the label is."
Administrator 1 guides participant's hand to the label.
"Please read to yourself what the label says."
Administrator 1 counts by hand/silently (one one thousand, two one thousand, etc.) to 10.

After 10 seconds, Administrator 1 verbalizes:
"Please tell me which labeled bin, #1 or #2, says XXXXX."

Administrator 2 starts stop-watch at the end of initial round of instructions for scoring purpose (i.e., only stop when activity has been completed or ended).

If participant declares being unable to do, or has not attempted/completed the task,
Give Level 1 prompt:
"Ok, [Participant's name]. Please try again. I am going to give you 2 labeled bins and ask you to tell me which one says XXXXXX."
Administrator 1 places labeled bin #1 in front of participant.
"I just put bin #1 in front of you. I will put your hand where the label is."
Administrator 1 guides participant's hand to the label.
"Please read to yourself what the label says."
Administrator 1 counts by hand/silently (one one thousand, two one thousand, etc.) to 10.

After 10 seconds, Administrator 1 places labeled bin #2 in front of participant.
Administrator 1 verbalizes:

"Now, I just put bin #2 in front of you. I will put your hand where the label is."

Administrator 1 guides participant's hand to the label.

"Please read to yourself what the label says."

Administrator 1 counts by hand/silently (one one thousand, two one thousand, etc.) to 10.

After 10 seconds, Administrator 1 verbalizes:

"Please tell me which labeled bin, #1 or #2, says XXXXX."

If participant declares being unable to do, then score as not completed and the duration as the maximum 180 seconds, and move to Item #1.

If participant has not attempted or appears to be trying and has not completed the task, give Level 2 prompt, which is the same as Level 1 prompt.

If participant has not attempted or appears to be trying and has not completed the task, give Level 3 prompt, which is the same as Level 1 prompt.

If participant has not completed the task, then score as not completed and the duration as the maximum 180 seconds. If participant appears still to be attempting to complete the task, Administrator 1 may elect to help the participant to complete the task though completion will not be scored.

Administrator 1 signals the score to Administrator 2 for confirmation.

Item #1a Recognize a school-related document

Administrator 1 signals test item number/letter and establishes eye contact with Administrator 2.

Administrator 1 places school document "XXXXX" in front of participant.

Administrator 1 verbalizes:

"For the next activity, I just put a piece of paper in front of you. I will put your hand where the paper is. Please read and tell me any word that is on the paper."

Administrator 2 starts stop-watch at the end of verbal instruction for 15 seconds.

Administrator 2 starts stop-watch at the end of verbal instruction for scoring purpose (i.e., only stop when activity has been completed or ended).

If participant declares being unable to do, or has not attempted/completed the task,

Give Level 1 prompt: "Ok, [Participant's name]. Please try one more time. Read and tell me any word that is on the paper."

Administrator 2 starts stop-watch at the end of verbal instruction for 15 seconds.

If participant declares being unable to do, then score Items #1a and b as not completed and the duration as the maximum 180 seconds, and move to Item #2.

If participant appears to be trying, then Administrator 1 follows the below prompts.

After 15 seconds,

If participant has not attempted/completed the task, give Level 2 prompt: "[Participant's name], please read and tell me any word that is on the paper."
Administrator 2 starts stop-watch at the end of verbal instruction for 15 seconds.

After 15 seconds,
If participant has not attempted/completed the task, give Level 3 prompt: "[Participant's name], please read and tell me any word that is on the paper."
Administrator 2 starts stop-watch at the end of verbal instruction for 15 seconds.

After 15 seconds,
If participant has not completed the task, then score Items #1a and b as not completed and the duration as the maximum 180 seconds, and move to Item #2. If participant appears still to be attempting to complete the task, Administrator 1 may elect to help the participant to complete the task though completion will not be scored.

Administrator 1 signals the score to Administrator 2 for confirmation.

Item #1b Identify the correct subject (asked by Administrator 1) of two school-related documents presented.

Administrator 1 signals test item number/letter and establishes eye contact with Administrator 2.

Administrator 1 verbalizes:
"For the next activity, I am going to give you 2 pieces of papers and ask you to tell me which one is about the subject XXXXX."

Administrator 1 places school document #1 in front of participant.
Administrator 1 verbalizes:
"I just put paper #1 in front of you. I will put your hand where the paper is. Please read it to yourself."
Administrator 2 starts stop-watch at the end of verbal instruction for 15 seconds.

After 15 seconds, Administrator 1 places school document #2 in front of participant.
Administrator 1 verbalizes:
"Now I put paper #2 in front of you. I will put your hand where the paper is. Please read it to yourself."
Administrator 2 starts stop-watch at the end of verbal instruction for 15 seconds.

After 15 seconds, Administrator 1 verbalizes:
Please tell me which paper, #1 or #2, is about the subject XXXXXX."

Administrator 2 starts stop-watch at the end of initial round of instructions for scoring purpose (i.e., only stop when activity has been completed or ended).

If participant declares being unable to do, or participant has not attempted/completed the task,
Give Level 1 prompt:
"Ok, [Participant's name]. Please try again. I am going to give you 2 pieces of papers and ask you to tell me which one is about the subject XXXXX."

Administrator 1 places school document #1 in front of participant.

Administrator 1 verbalizes:
"I just put paper #1 in front of you. Please read it to yourself."
Administrator 2 starts stop-watch at the end of verbal instruction for 15 seconds.

After 15 seconds, Administrator 1 places school document #2 in front of participant.
Administrator 1 verbalizes:
"Now I put paper #2 in front of you. Please read it to yourself."
Administrator 2 starts stop-watch at the end of verbal instruction for 15 seconds.

After 15 seconds, Administrator 1 verbalizes:
"Please tell me which paper, #1 or #2, is about the subject XXXXXX."

If participant declares being unable to do, then score as not completed and the duration as the maximum 180 seconds, and move to Item #2.

If participant has not attempted or appears to be trying and has not completed the task, give Level 2 prompt, which is the same as Level 1 prompt.

If participant has not attempted or appears to be trying and has not completed the task, give Level 3 prompt, which is the same as Level 1 prompt.

If participant has not completed the task, score as not completed and the duration as the maximum 180 seconds, and move to Item #2. If participant appears still to be attempting to complete the task, Administrator 1 may elect to help the participant to complete the task though completion will not be scored.

Administrator 1 signals the score to Administrator 2 for confirmation.

Item #2a Recognize options from a printed Menu.

Administrator 1 signals test item number/letter and establishes eye contact with Administrator 2.

Administrator 1 places the menu in front of participant.
Administrator 1 verbalizes:
"For the next activity, I just put a menu in front of you. I will put your hand where the menu is. Please, read and tell me any word on the menu."
Administrator 2 starts stop-watch at the end of verbal instruction for 15 seconds.

Administrator 2 starts stop-watch at the end of verbal instruction for scoring purpose (i.e., only stop when activity has been completed or ended).

If participant declares being unable to do, or participant has not attempted/completed the task,
Give Level 1 prompt:
"Ok, [Participant's name]. Please try one more time. Read and tell me any word that is on the menu."
Administrator 2 starts stop-watch at the end of verbal instruction for 15 seconds.

If participant declares being unable to do, then score Items #2a and b as not completed and the duration as the maximum 180 seconds, and move to Item #5.

If participant appears to be trying, then Administrator 1 follows the below prompts.
After 15 seconds,
If participant has not attempted/completed the task, give Level 2 prompt: "[Participant's name], please read and tell me any word on the menu."
Administrator 2 starts stop-watch at the end of verbal instruction for 15 seconds.

After 15 seconds,
If participant has not attempted/completed the task, give Level 3 prompt: "[Participant's name], please read and tell me any word on the menu."
Administrator 2 starts stop-watch at the end of verbal instruction for 15 seconds.

After 15 seconds,
If participant has not completed the task, then score Items #2a and b as not completed and the duration as the maximum 180 seconds, and move to Item #5. If participant appears still to be attempting to complete the task, Administrator 1 may elect to help the participant to complete the task though completion will not be scored.

Administrator 1 signals the score to Administrator 2 for confirmation.

Item #2b Select food item from a menu.

Administrator 1 signals test item number/letter and establishes eye contact with Administrator 2.

Administrator 1 places the menu in front of participant.
Administrator 1 verbalizes:
"Now [Participant's name], please tell me what you would want to order from the menu."
Administrator 2 starts stop-watch at the end of verbal instruction for 15 seconds.

Administrator 2 starts stop-watch at the end of verbal instruction for scoring purpose (i.e., only stop when activity has been completed or ended).

If participant declares being unable to do, or after 15 seconds and participant has not attempted/completed the task,
Give Level 1 prompt: "Ok, [Participant's name], please try again and tell me what you would want to order from the menu."
Administrator 2 starts stop-watch at the end of verbal instruction for 15 seconds.

If participant declares being unable to do, then score Items #2a and b as not completed and the duration as the maximum 180 seconds, and move to Item #5.

After 15 seconds,
If participant has not attempted or appears to be trying and has not completed the task, give Level 2 prompt: "[Participant's name], please tell me what you would want to order from the menu."
Administrator 2 starts stop-watch at the end of verbal instruction for 15 seconds.

After 15 seconds,
If participant has not attempted or appears to be trying and has not completed the task, give Level 3 prompt: "[Participant's name], please tell me what you would want to order from the menu."

Administrator 2 starts stop-watch at the end of verbal instruction for 15 seconds.

After 15 seconds,

If participant has not completed the task, then score as not completed and the duration as the maximum 180 seconds, and move to Item #5. If participant appears still to be attempting to complete the task, Administrator 1 may elect to help the participant to complete the task though completion will not be scored.

Administrator 1 signals the score to Administrator 2 for confirmation.

Item #5a Recognize text on a page in a book.

Administrator 1 signals test item number/letter and establishes eye contact with Administrator 2.

Administrator 1 places a children's book (preferably without pictures), opened to a page (the content of which is familiar to the Administrator 1) in front of participant.
Administrator 1 verbalizes:

"For the next activity, I just put a book page in front of you. I will put your hand where the book page is. Please read and tell me any word on the page."

Administrator 2 starts stop-watch at the end of verbal instruction for 15 seconds.

Administrator 2 starts stop-watch at the end of verbal instruction for scoring purpose (i.e., only stop when activity has been completed or ended).

If participant declares being unable to do, or after 15 seconds and participant has not attempted/completed the task,

Give Level 1 prompt: "Ok, [Participant's name]. Please try one more time. Read and tell me any word on the page."

Administrator 2 starts stop-watch at the end of verbal instruction for 15 seconds.

If participant declares being unable to do, then score Items #5a and b as not completed and the duration as the maximum 180 seconds, and move to Item #6.

If participant appears to be trying, then Administrator 1 follows the below prompts.
After 15 seconds,

If participant has not attempted/completed the task, give Level 1 prompt: "[Participant's name], please read and tell me any word on the page."

After 15 seconds,

If participant has not attempted/completed the task, give Level 2 prompt: "[Participant's name], please read and tell me any word on the page."

Administrator 2 starts stop-watch at the end of verbal instruction for 15 seconds.

After 15 seconds,

If participant has not attempted/completed the task, give Level 3 prompt: "[Participant's name], please read and tell me any word on the page."

Administrator 2 starts stop-watch at the end of verbal instruction for 15 seconds.

After 15 seconds,

If participant declares being unable to do, then score Items #5a and b as not completed and the duration as the maximum 180 seconds, and move to Item #6. If participant appears still to be attempting to complete the task, Administrator 1 may elect to help the participant to complete the task though completion will not be scored.

Administrator 1 signals the score to Administrator 2 for confirmation.

Item #5b Answer specific question relating to content from a page of a book.

Administrator 1 signals test item number/letter and establishes eye contact with Administrator 2.

Administrator 1 places a children's book (preferably without pictures), opened to a page (the content of which is familiar to the Administrator 1) in front of participant. Administrator 1 can elect to use the same set up as in #5a.
Administrator 1 verbalizes:
"Now from this page of the book, please read and tell me XXXXXX (e.g., what was the best friend's name? Or, what did Robbie lose?).
Administrator 2 starts stop-watch at the end of verbal instruction for 15 seconds.

Administrator 2 starts stop-watch at the end of verbal instruction for scoring purpose (i.e., only stop when activity has been completed or ended).

If participant declares being unable to do, or after 15 seconds and participant has not attempted/completed the task,
Give Level 1 prompt: "Ok, [Participant's name]. Please try again. Read and tell me XXXXXX (e.g., what was the best friend's name? Or, what did Robbie lose?)."
Administrator 2 starts stop-watch at the end of verbal instruction for 15 seconds.

If participant declares being unable to do, then score as not completed and the duration as the maximum 180 seconds, and move to Item #6.

After 15 seconds,
If participant has not attempted or appears to be trying and has not completed the task, give Level 2 prompt: "[Participant's name], please read and tell me XXXXXX (e.g., what was the best friend's name? Or, what did Robbie lose?)."
Administrator 2 starts stop-watch at the end of verbal instruction for 15 seconds.

After 15 seconds,
If participant has not attempted or appears to be trying and has not completed the task, give Level 3 prompt: "[Participant's name], please read and tell me XXXXXX (e.g., what was the best friend's name? Or, what did Robbie lose?)."
Administrator 2 starts stop-watch at the end of verbal instruction for 15 seconds.

After 15 seconds,
If participant has not completed the task, then score as not completed and the duration as the maximum 180 seconds, and move to Item #6. If participant appears still to be attempting to complete the task, Administrator 1 may elect to help the participant to complete the task though completion will not be scored.

Administrator 1 signals the score to Administrator 2 for confirmation.

Item #6a Recognize the color of a uniformed, single, primary colored item.

Administrator 1 signals test item number/letter and establishes eye contact with Administrator 2.

Administrator 1 places a piece (~10x10 cm or larger) of paper of a primary color (red, yellow, or blue) in front of participant.
Administrator 1 verbalizes:
"For this next activity, I will put your hand on a piece of paper in front of you. Please tell me the color of the piece of paper."
Administrator 2 starts stop-watch at the end of verbal instruction for 15 seconds.

Administrator 2 starts stop-watch at the end of verbal instruction for scoring purpose (i.e., only stop when activity has been completed or ended).

If participant declares being unable to do, or after 15 seconds and participant has not attempted/completed the task,
Give Level 1 prompt: "Ok, [Participant's name]. Please try one more time and tell me the color of the piece of paper in front of you."
Administrator 2 starts stop-watch at the end of verbal instruction for 15 seconds.

If participant declares being unable to do, then score Items #6a and b as not completed and the duration as the maximum 180 seconds, and move to Item #3.

If participant appears to be trying, then Administrator 1 follows the below prompts.

After 15 seconds,
If participant has not attempted/completed the task, give Level 2 prompt: "[Participant's name], please tell me the color of the piece of paper in front of you."
Administrator 2 starts stop-watch at the end of verbal instruction for 15 seconds.

After 15 seconds,
If participant has not attempted/completed the task, give Level 3 prompt: "[Participant's name], please tell me the color of the piece of paper in front of you."
Administrator 2 starts stop-watch at the end of verbal instruction for 15 seconds.

After 15 seconds,
If participant has not completed the task, Administrator 1 scores Items #6a and b as not completed and the duration as the maximum 180 seconds, and move to Item #3. If participant appears still to be attempting to complete the task, Administrator 1 may elect to help the participant to complete the task though completion will not be scored.

Administrator 1 signals the score to Administrator 2 for confirmation.

Item #6b Recognize one of two colored items.

Administrator 1 signals test item number/letter and establishes eye contact with Administrator 2.

Administrator 1 verbalizes:
"For the next activity, I am going to give you 2 colored papers and ask you to tell me which one has the color XXXXXX."
Administrator 1 places colored paper #1 in front of participant.
"I just put colored paper #1 in front of you. I will put your hand where the paper is. Please find out for yourself what color it is."
Administrator 1 counts by hand/silently at the end of verbal instruction for 10 seconds.

After 10 seconds, Administrator 1 places colored paper #2 in front of participant.
Administrator 1 verbalizes:
"Now I just put colored paper #2 in front of you. I will put your hand where the paper is. Please find out for yourself what color it is."
Administrator 1 counts by hand/silently at the end of verbal instruction for 10 seconds.

After 10 seconds, Administrator 1 verbalizes:
"Please tell me which paper, #1 or #2, has the color XXXXXX."

Administrator 2 starts stop-watch at the end of initial round of instructions for scoring purpose (i.e., only stop when activity has been completed or ended).

If participant declares being unable to do, or after 15 seconds and participant has not attempted/completed the task,
Give Level 1 prompt:
"Ok, [Participant's name]. Please try again. I am going to give you 2 colored papers and ask you to tell me which one has the color XXXXXX."
Administrator 1 places colored paper #1 in front of participant.
"I just put colored paper #1 in front of you. Please find out for yourself what color it is."
Administrator 1 counts by hand/silently at the end of verbal instruction for 10 seconds.

After 10 seconds, Administrator 1 places colored paper #2 in front of participant.
Administrator 1 verbalizes:
"Now I just put colored paper #2 in front of you. Please find out for yourself what color it is."
Administrator 1 counts by hand/silently at the end of verbal instruction for 10 seconds.

After 10 seconds, Administrator 1 verbalizes:
"Please tell me which paper, #1 or #2, has the color XXXXXX."

After 15 seconds,
If participant has not attempted or appears to be trying and has not completed the task, give Level 2 prompt, which is the same as Level 1 prompt.

After 15 seconds,
If participant has not attempted or appears to be trying and has not completed the task, give Level 2 prompt, which is the same as Level 1 prompt.

After 15 seconds,
If participant has not completed the task, then score as not completed and the duration as the maximum 180 seconds, and move to Item #3. If participant appears still to be attempting to complete the task, Administrator 1 may elect to help the participant to complete the task though completion will not be scored.

Administrator 1 signals the score to Administrator 2 for confirmation.

Item #3a Recognize a room sign.

Administrator 1 signals test item number/letter and establishes eye contact with Administrator 2.

Administrator 1 verbalizes:
"For the next activity, we are going to go outside of the room. I am going to ask you about signs."
Administrator 1 positions participant in front of a room sign (e.g., room number, bathroom sign), within 2 feet of the sign. Administrator 1 verbalizes:
"You are standing in front of a sign for the room. I will put your hand where the sign is. Please read and tell me what the sign says."
Administrator 2 starts stop-watch at the end of verbal instruction for 15 seconds.

Administrator 2 starts stop-watch at the end of verbal instruction for scoring purpose (i.e., only stop when activity has been completed or ended).

If participant declares being unable to do, or after 15 seconds and participant has not attempted/completed the task,
Give Level 1 prompt: "Ok, [Participant's name]. Please try one more time. Read and tell me what the sign says."
Administrator 2 starts stop-watch at the end of verbal instruction for 15 seconds.

If participant declares being unable to do, then score Items #3a and b as not completed and the duration as the maximum 180 seconds, and move to Item #7.

If participant appears to be trying, then Administrator 1 follows the below prompts.
After 15 seconds,
If participant has not attempted/completed the task, give Level 2 prompt: "[Participant's name], please read and tell me what the sign says."
Administrator 2 starts stop-watch at the end of verbal instruction for 15 seconds.

After 15 seconds,
If participant has not attempted/completed the task, give Level 3 prompt: "[Participant's name], please read and tell me what the sign says."
Administrator 2 starts stop-watch at the end of verbal instruction for 15 seconds.

After 15 seconds,
If participant has not completed the task, then score Items #3a and b as not completed and the duration as the maximum 180 seconds, and move to Item #7. If participant appears still to be attempting to complete the task, Administrator 1 may elect to help the participant to complete the task though completion will not be scored.

Item #3b Identify which room to enter based on need/preference.

Administrator 1 signals test item number/letter and establishes eye contact with Administrator 2.

Administrator 1 verbalizes:
"For the next activity, I am going to ask you to choose between two room signs."
Administrator 1 places participant in front of sign #1, within 2 feet of the sign.
"You are in front of room sign #1. Please read to yourself what the sign says."
Administrator 1 counts by hand/silently (one one thousand, two one thousand, etc.) to 10.

After 10 seconds, Administrator 1 places participant in front of sign #2, within 2 feet of the sign.
Administrator 1 verbalizes:
"You are in front of room sign #2. Please read to yourself what the sign says."
Administrator 1 counts by hand/silently (one one thousand, two one thousand, etc.) to 10.

After 10 seconds, Administrator 1 verbalizes:
"Please tell me which room, #1 or #2, you would go in to 'XXXXX' (e.g., cook food, use the restroom, etc.)"

Administrator 2 starts stop-watch at the end of initial round of instructions for scoring purpose (i.e., only stop when activity has been completed or ended).

If participant declares being unable to do, or has not attempted/completed the task,
Give Level 1 prompt:
"Ok, [Participant's name]. Please try again. I am going to ask you to choose between two room signs."
Administrator 1 places participant in front of sign #1, within 2 feet of the sign.
"You are in front of room sign #1. Please read to yourself what the sign says."
Administrator 1 counts by hand/silently (one one thousand, two one thousand, etc.) to 10.

After 10 seconds, Administrator 1 places participant in front of sign #2, within 2 feet of the sign. Administrator 1 verbalizes:
"You are in front of room sign #2. Please read to yourself what the sign says."

After 10 seconds, Administrator 1 verbalizes:
"Please tell me which room, #1 or #2, you would go in to 'XXXXX' (e.g., cook food, use the restroom, etc.)"

If participant declares being unable to do, then score as not completed and the duration as the maximum 180 seconds, and move to Item #7.

If participant has not attempted or appears to be trying and has not completed the task, give Level 2 prompt, which is the same as Level 1 prompt.

If participant has not attempted or appears to be trying and has not completed the task, give Level 3 prompt, which is the same as Level 1 prompt.

If participant has not completed the task, then score as not completed and the duration as the maximum 180 seconds, and move to Item #7. If participant appears still to be attempting to complete the task, Administrator 1 may elect to help the participant to complete the task though completion will not be scored.

Administrator 1 signals the score to Administrator 2 for confirmation.

Item #7a Recognize a facing person with prompting.

Administrator 1 signals test item number/letter and establishes eye contact with Administrator 2. A study team member, different from the Administrator 1, positions (~6 ft) in front of participant before verbal instructions provided by the Administrator 1. Administrator 1 moves out of participant's field of vision.

Administrator 1 verbalizes:
"For the next activity, I am going to ask you about the person in front of you. Please tell me who that person is."
Administrator 2 starts stop-watch at the end of verbal instruction for 15 seconds.

Administrator 2 starts stop-watch at the end of verbal instruction for scoring purpose (i.e., only stop when activity has been completed or ended).

If participant declares being unable to do, or has not attempted/completed the task,
Give Level 1 prompt: "Ok, [Participant's name]. Please try one more time and tell me who is the person in front of you."
Administrator 2 starts stop-watch at the end of verbal instruction for 15 seconds.

If participant declares being unable to do, then score Items #7a and b as not completed and the duration as the maximum 180 seconds, and move to Item #8 or end the Function test (e.g., participant appears fatigued or inattentive).

After 15 seconds,
If participant has not attempted/completed the task, give Level 2 prompt: "[Participant's name], I am going to ask you about the person in front of you. Please tell me who that person is."
Administrator 2 starts stop-watch at the end of verbal instruction for 15 seconds.

After 15 seconds,
If participant has not attempted/completed the task, give Level 3 prompt: "[Participant's name], I am going to ask you about the person in front of you. Please tell me who that person is."
Administrator 2 starts stop-watch at the end of verbal instruction for 15 seconds.

After 15 seconds,
If participant has not completed the task, then score Items #7a and b as not completed and the duration as the maximum 180 seconds, and move to Item #8 or end the Function Test (e.g., participant appears fatigued or inattentive). If participant appears still to be attempting to complete the task, Administrator 1 may elect to help the participant to complete the task though completion will not be scored.

Administrator 1 signals the score to Administrator 2 for confirmation.

Item #7b Recognize a facing person without prompt.

Administrator 1 signals test item number/letter and establishes eye contact with Administrator 2.

A study team member, different from the Administrator 1, positions (~6 ft) in front of participant before, during, or after verbal instructions given by the Administrator 1. Administrator 1 moves out of participant's field of vision.
Verbal instruction to be provided by the Administrator 1:

“In the next few minutes, there will be another person in front of you. Please tell me who that person is whenever you see them.”

Administrator 2 starts stop-watch at the end of verbal instruction for 15 seconds.

Administrator 2 starts stop-watch at the end of verbal instruction for scoring purpose (i.e., only stop when activity has been completed or ended).

If participant declares being unable to do, or after 15 seconds and participant has not attempted/completed the task, give Level 1 prompt: “Ok, [Participant's name]. Please try again. In the next few minutes, there will be another person in front of you. Please tell me who that person is whenever you see them.”

If participant declares being unable to do, then score as not completed and the duration as the maximum 180 seconds, and move to Item #8 or end the Function test (e.g., participant appears fatigued or inattentive).

After 15 seconds,

If participant has not attempted or appears to be trying and has not completed the task, give Level 2 prompt: “[Participant's name]. In the next few minutes, there will be another person in front of you. Please tell me who that person is whenever you see them.”

After 15 seconds,

If participant has not attempted or appears to be trying and has not completed the task, give Level 3 prompt: “[Participant's name]. In the next few minutes, there will be another person in front of you. Please tell me who that person is whenever you see them.”

After 15 seconds,

If participant declares being unable to do, then score as not completed and the duration as the maximum 180 seconds, and move to Item #8 or end the Function test (e.g., participant appears fatigued or inattentive). If participant appears still to be attempting to complete the task, Administrator 1 may elect to help the participant to complete the task though completion will not be scored.

Administrator 1 signals the score to Administrator 2 for confirmation.

If **proceeding** with exploratory items, Administrator 1 verbalizes:

“Let's go back to the room.”

If **not proceeding** with exploratory items (e.g., participant is fatigued or inattentive, or if time is running out), Administrator 1 verbalizes:

“This is the end of these activities. Thank you for working with me. Let's go back to the room.”

Item #8a Report the day and date.

Administrator 1 signals test item number/letter and establishes eye contact with Administrator 2.

Administrator 1 verbalizes:
“Please tell me today’s date.”
Administrator 2 starts stop-watch at the end of verbal instruction for 15 seconds.

Administrator 2 starts stop-watch at the end of verbal instruction for scoring purpose (i.e., only stop when activity has been completed or ended).

If participant declares being unable to do, or after 15 seconds and participant has not attempted/completed the task, give Level 1 prompt: “Ok, [Participant’s name]. Please try one more time and tell me today’s date.”
Administrator 2 starts stop-watch at the end of verbal instruction for 15 seconds.

If participant declares being unable to do, then score Items #8a and b as not completed and the duration as the maximum 180 seconds, and move to Item #9.

After 15 seconds,
If participant has not attempted/completed the task, give Level 2 prompt: “[Participant’s name], please tell me today’s date.”
Administrator 2 starts stop-watch at the end of verbal instruction for 15 seconds.

After 15 seconds,
If participant has not attempted/completed the task, give Level 3 prompt: “[Participant’s name], please tell me today’s date.”
Administrator 2 starts stop-watch at the end of verbal instruction for 15 seconds.

After 15 seconds,
If participant declares being unable to do, then score Items #8a and b as not completed and the duration as the maximum 180 seconds, and move to Item #9. If participant appears still to be attempting to complete the task, Administrator 1 may elect to help the participant to complete the task though completion will not be scored.

Administrator 1 signals the score to Administrator 2 for confirmation.

Item #8b Identify the day and answer test admin’s question (how many days until Sunday)

Administrator 1 signals test item number/letter and establishes eye contact with Administrator 2.

Administrator 1 verbalizes:
“Please tell me what day of the week is today, and how many days until Sunday.”
Administrator 2 starts stop-watch at the end of verbal instruction for 15 seconds.

Administrator 2 starts stop-watch at the end of verbal instruction for scoring purpose (i.e., only stop when activity has been completed or ended).

If participant declares being unable to do, or after 15 seconds and participant has not attempted/completed the task, give Level 1 prompt: “Ok, [Participant’s name]. Please try again and tell me what day of the week is today, and how many days until Sunday.”
Administrator 2 starts stop-watch at the end of verbal instruction for 15 seconds.

If participant declares being unable to do, then score as not completed and the duration as the maximum 180 seconds, and move to Item #9.

After 15 seconds,

If participant has not attempted or appears to be trying and has not completed the task, give Level 2 prompt: "[Participant's name], please tell me what day of the week is today, and how many days until Sunday."

Administrator 2 starts stop-watch at the end of verbal instruction for 15 seconds.

After 15 seconds,

If participant has not attempted or appears to be trying and has not completed the task, give Level 3 prompt: "[Participant's name], please tell me what day of the week is today, and how many days until Sunday."

Administrator 2 starts stop-watch at the end of verbal instruction for 15 seconds.

After 15 seconds,

If participant declares being unable to do, then score as not completed and the duration as the maximum 180 seconds, and move to Item #9. If participant appears still to be attempting to complete the task, Administrator 1 may elect to help the participant to complete the task though completion will not be scored.

Administrator 1 signals the score to Administrator 2 for confirmation.

Item #9a Recognize a snack product by its label.

Administrator 1 signals test item number/letter and establishes eye contact with Administrator 2.

Administrator 1 places a bag of snack (e.g., Goldfish crackers, Teddy Grahams, pretzels) in front of study participant. Verbalizes:

"For the next activity, I am going to ask you about snack labels. I will put or hold a snack bag/container in front of you. Please read and tell me the name of the snack bag/container."

Administrator 2 starts stop-watch at the end of verbal instruction for 15 seconds.

Administrator 2 starts stop-watch at the end of verbal instruction for scoring purpose (i.e., only stop when activity has been completed or ended).

If participant declares being unable to do, or after 15 seconds and participant has not attempted/completed the task, give Level 1 prompt:

"Ok, [Participant's name]. Please try one more time. Read and tell me the name of the snack I placed in front of you."

Administrator 2 starts stop-watch at the end of verbal instruction for 15 seconds.

If participant declares being unable to do, then score Items #9a and b as not completed and the duration as the maximum 180 seconds, and move to Item #10.

After 15 seconds,

If participant has not attempted/completed the task, give Level 2 prompt: "[Participant's name], please read and tell me the name of the snack bag I placed in front of you."

Administrator 2 starts stop-watch at the end of verbal instruction for 15 seconds.

After 15 seconds,
If participant has not attempted/completed the task, give Level 3 prompt: "[Participant's name], please read and tell me the name of the snack bag I placed in front of you."
Administrator 2 starts stop-watch at the end of verbal instruction for 15 seconds.

After 15 seconds,
If participant has not completed the task, then score Items #9a and b as not completed and the duration as the maximum 180 seconds, and move to Item #10. If participant appears still to be attempting to complete the task, Administrator 1 may elect to help the participant to complete the task though completion will not be scored.

Administrator 1 signals the score to Administrator 2 for confirmation.

Item #9b Recognize and take the preferred snack option.

Administrator 1 signals test item number/letter and establishes eye contact with Administrator 2.

Administrator 1 verbalizes:
"For the next activity, I am going to ask you to choose from two snacks."
Administrator 1 places snack bag #1 (e.g. Goldfish crackers, Teddy Grahams, pretzels) in front of participant.
"Snack #1 is in front of you. Please read to yourself what it is."
Administrator 1 counts by hand/silently at the end of verbal instruction for 10 seconds.

After 10 seconds, Administrator 1 places snack #2 in front of participant.
Administrator 1 verbalizes:
"Snack #2 is in front of you. Please read to yourself what it is."
Administrator 1 counts by hand/silently at the end of verbal instruction for 10 seconds.

After 10 seconds, Administrator 1 verbalizes:
"Please tell me which snack, #1 or #2, you prefer."

Administrator 2 starts stop-watch at the end of initial round of instructions for scoring purpose (i.e., only stop when activity has been completed or ended).

If participant declares being unable to do, or has not attempted/completed the task,
Give Level 1 prompt:
"Ok, [Participant's name]. Please try again. I am going to ask you to choose from two snacks."
Administrator 1 places snack bag #1 (e.g. Goldfish crackers, Teddy Grahams, pretzels) in front of participant.
"Snack #1 is in front of you. Please read to yourself what it is."
Administrator 1 counts by hand/silently at the end of verbal instruction for 10 seconds.

After 10 seconds, Administrator 1 places snack #2 in front of participant.
Administrator 1 verbalizes:
"Snack #2 is in front of you. Please read to yourself what it is."
Administrator 1 counts by hand/silently at the end of verbal instruction for 10 seconds.

After 10 seconds, Administrator 1 verbalizes:
"Please tell me which snack, #1 or #2, you prefer."

If participant declares being unable to do, then score as not completed and the duration as the maximum 180 seconds, and move to Item #10.

After 15 seconds,
If participant has not attempted or appears to be trying and has not completed the task, give Level 2 prompt, which is the same as Level 1 prompt.

After 15 seconds,
If participant has not attempted or appears to be trying and has not completed the task, give Level 3 prompt, which is the same as Level 1 prompt.

After 15 seconds,
If participant declares being unable to do, then score as not completed and the duration as the maximum 180 seconds, and move to Item #10. If participant appears still to be attempting to complete the task, Administrator 1 may elect to help the participant to complete the task though completion will not be scored.

Administrator 1 signals the score to Administrator 2 for confirmation.

Item #10a Recognize options from a Menu on a screen device.

Administrator 1 signals test item number/letter and establishes eye contact with Administrator 2.

Administrator 1 displays a menu on a screen device.
Administrator 1 verbalizes:
"For the next activity, I am going to ask you about a list of items. I just put a list on a(n) XXXXXX (e.g., iPad, computer, etc.) in front of you. I will put your hand where the computer is. Please read and tell me any word on the list."
Administrator 2 starts stop-watch at the end of verbal instruction for 15 seconds.

Administrator 2 starts stop-watch at the end of verbal instruction for scoring purpose (i.e., only stop when activity has been completed or ended).

If participant declares being unable to do, or has not attempted/completed the task,
Give Level 1 prompt:
"Ok, [Participant's name]. Please try one more time. Read and tell me any word on the list."
Administrator 2 starts stop-watch at the end of verbal instruction for 15 seconds.

If participant declares being unable to see, then score Items #10a and b as not completed and the duration as the maximum 180 seconds, and end the Function Test.

If participant appears to be trying, then Administrator 1 follows the below prompts.

After 15 seconds,
If participant has not attempted/completed the task, give Level 2 prompt: "[Participant's name], please read and tell me any word on the list."
Administrator 2 starts stop-watch at the end of verbal instruction for 15 seconds.

After 15 seconds,

If participant has not attempted/completed the task, give Level 3 prompt: “[Participant’s name], please read and tell me any word on the list.”

Administrator 2 starts stop-watch at the end of verbal instruction for 15 seconds.

After 15 seconds,

If participant has not completed the task, then score Items #10a and b as not completed and the duration as the maximum 180 seconds, and end the Function Test. If participant appears still to be attempting to complete the task, Administrator 1 may elect to help the participant to complete the task though completion will not be scored.

Administrator 1 signals the score to Administrator 2 for confirmation.

Item #10b Recognize options from a Menu list and decide whether an action can be done.

Administrator 1 signals test item number/letter and establishes eye contact with Administrator 2.

Administrator 1 displays a list on a screen device. Administrator 1 may elect to use the same set-up in #10a.

Administrator 1 verbalizes:

“For the next activity, I am going to ask you whether an item is on the list. Please read and tell me whether you can XXXXXX (e.g., play song XXXXXX? Or, do XXXXXX).”

Administrator 2 starts stop-watch at the end of verbal instruction for 15 seconds.

Administrator 2 starts stop-watch at the end of verbal instruction for scoring purpose (i.e., only stop when activity has been completed or ended).

If participant declares being unable to do, or after 15 seconds and participant has not attempted/completed the task, give Level 1 prompt: “Ok, [Participant’s name]. Please try again. I am going to ask you whether an item is on the list. Please read and tell me whether you can XXXXXX (e.g., play song XXXXXX? Or, do XXXXXX)?”

Administrator 2 starts stop-watch at the end of verbal instruction for 15 seconds.

If participant declares being unable to see, then score Items as not completed and the duration as the maximum 180 seconds, and end the Function Test.

After 15 seconds,

If participant has not attempted or appears to be trying and has not completed the task, give Level 2 prompt, which is the same as Level 1 prompt.

Administrator 2 starts stop-watch at the end of verbal instruction for 15 seconds.

After 15 seconds,

If participant has not attempted or appears to be trying and has not completed the task, give Level 3 prompt, which is the same as Level 1 prompt.

Administrator 2 starts stop-watch at the end of verbal instruction for 15 seconds.

After 15 seconds,

If participant declares being unable to see, then score not completed and the duration as the maximum 180 seconds, and end the Function Test. If participant appears still to be attempting to complete the task, Administrator 1 may elect to help the participant to complete the task though completion will not be scored.

Administrator 1 signals the score to Administrator 2 for confirmation.

Administrator 1 verbalizes:

"This is the end of these activities. Thank you for working with me. You may relax."

Administrator 1 verbalizes:

"Now, I would like to learn how the OrCam helps you do some activities we tried before. These are things that you would normally do every day. I will ask you to do these things by yourself using the OrCam, without help from your parents. Some of these things you will be able to do, and some you may not be able to do by yourself. That's ok! You can try a few times. Please do your best.

Your parent and other people will watch and take notes. They will stay quiet during our activities. Before you start, do you have any question?

Please put on the eyeglasses with the OrCam attached. Remember to look straight ahead, keep your head still, and listen carefully to what the OrCam says. Okay, let's begin."

Item #4a Recognize a printed label on a bin/drawer.

Administrator 1 signals test item number/letter and establishes eye contact with Administrator 2. Administrator 1 places a labeled bin in front of participant. The bin is labeled with single-word items (e.g., "shirts", "pants", "socks") in printed, capital letters, 1-inch in height (Helvetica font, size 72), using black-ink on standard white paper. Administrator 1 checks that blue light on OrCam is on. If not, Administrator 1 taps/asks participant to tap OrCam once to wake up the OrCam.

Administrator 1 verbalizes:

"For this activity, I am going to ask you about labels. I just put a labeled bin in front of you. I will put your hand where the label is."

Administrator 1 guides participant's hand to the label.

"Please read and tell me what the label says."

Administrator 2 starts stop-watch at the end of verbal instruction for 15 seconds.

Administrator 2 starts stop-watch at the end of verbal instruction for scoring purpose (i.e., only stop when activity has been completed or ended).

After 15 seconds,

If participant has not attempted/completed the task, give Level 1 prompt:

"[Participant's name], use the OrCam on your eyeglasses like you did in practice. Please read and tell me what the label says." Administrator 2 starts stop-watch at the end of verbal instruction for 15 seconds.

After 15 seconds,

If participant has not attempted/completed the task, give Level 2 prompt:

verbal reminders to use the device and verbal instructions on how to use the device, "[Participant's name], please read and tell me what the label says." Administrator 2 starts stop-watch at the end of verbal instruction for 15 seconds.

After 15 seconds,

If participant has not attempted/completed the task, give Level 3 prompt:

verbal and non-verbal reminder to use the device (e.g., adjusting the participant's head/finger to aim/point at the object in question),
"[Participant's name], please read and tell me what the label says." Administrator 2 starts stop-watch at the end of verbal instruction for 15 seconds.

After 15 seconds,
If participant declares being unable to do, then score as not completed and the duration as the maximum 180 seconds, and move to Item #4b. If participant appears still to be attempting to complete the task, Administrator 1 may elect to help the participant to complete the task though completion will not be scored.

Administrator 1 signals the score to Administrator 2 for confirmation.

Item #4b Recognize one of two bin/drawer labels and select the appropriate bin/drawer as directed by the examiner.

Administrator 1 signals test item number/letter and establishes eye contact with Administrator 2.

Administrator 1 checks that blue light on OrCam is on. If not, Administrator 1 taps/asks participant to tap OrCam once to wake up the OrCam. Verbalizes:
"For the next activity, I am going to give you 2 labeled bins and ask you to tell me which one says XXXXXX."
Administrator 1 places labeled bin #1 in front of participant.
"I just put bin #1 in front of you. I will put your hand where the label is."
Administrator 1 guides participant's hand to the label.
"Please read to yourself what the label says."
Administrator 1 counts by hand/silently at the end of verbal instruction for 10 seconds.

After 10 seconds, Administrator 1 places labeled bin #2 in front of participant.
Administrator 1 verbalizes:
"Now, I just put bin #2 in front of you. I will put your hand where the label is."
Administrator 1 guides participant's hand to the label.
"Please read to yourself what the label says."
Administrator 1 counts by hand/silently at the end of verbal instruction for 10 seconds.

After 10 seconds, Administrator 1 verbalizes:
"Please tell me which labeled bin, #1 or #2, says XXXXX."

Administrator 2 starts stop-watch at the end of initial round of instructions for scoring purpose (i.e., only stop when activity has been completed or ended).

If participant has not attempted/completed the task
Give Level 1 prompt:
"[Participant's name], use the OrCam on your eyeglasses like you did in practice. I am going to give you 2 labeled bins and ask you to tell me which one says XXXXXX."
Administrator 1 places labeled bin #1 in front of participant.
"I just put bin #1 in front of you. I will put your hand where the label is."
Administrator 1 guides participant's hand to the label.
"Please read to yourself what the label says."

Administrator 1 counts by hand/silently at the end of verbal instruction for 10 seconds.

After 10 seconds, Administrator 1 places labeled bin #2 in front of participant.
Administrator 1 verbalizes:
"Now, I just put bin #2 in front of you. I will put your hand where the label is."
Administrator 1 guides participant's hand to the label.
"Please read to yourself what the label says."
Administrator 1 counts by hand/silently at the end of verbal instruction for 10 seconds.

After 10 seconds, Administrator 1 verbalizes:
"Please tell me which labeled bin, #1 or #2, says XXXXX."

If participant has not attempted/completed the task
Give Level 2 prompt:
verbal reminders to use the device and verbal instructions on how to use the device,
round of task instructions exactly as in Level 1 prompt

If participant has not attempted/completed the task
Give Level 3 prompt:
verbal and non-verbal reminder to use the device (e.g., adjusting the participant's head/finger to aim/point at the object in question),
round of task instructions exactly as in Level 1 prompt

If participant declares being unable to do, then score as not completed and the duration as the maximum 180 seconds, and move to Item #1a. If participant appears still to be attempting to complete the task, Administrator 1 may elect to help the participant to complete the task though completion will not be scored.

Administrator 1 signals the score to Administrator 2 for confirmation.

Item #1a Recognize a school-related document

Administrator 1 signals test item number/letter and establishes eye contact with Administrator 2.

Administrator 1 places school document "XXXXX" in front of participant. Administrator 1 checks that blue light on OrCam is on. If not, Administrator 1 taps/asks participant to tap OrCam once to wake up the OrCam.
Administrator 1 verbalizes:
"For this activity, I just put a piece of paper in front of you. I will put your hand where the paper is. Please read and tell me any word that is on the paper."
Administrator 1 counts by hand/silently at the end of verbal instruction for 10 seconds and stop the OrCam from reading (if needed). Verbalizes: "Please tell me any word on the paper."

Administrator 2 starts stop-watch at the end of verbal instruction for scoring purpose (i.e., only stop when activity has been completed or ended).

If participant has not attempted/completed the task, give Level 1 prompt:

“[Participant’s name], use the OrCam on your eyeglasses like you did in practice. Please read and tell me any word on the paper in front of you.” Administrator 2 starts stop-watch at the end of verbal instruction for 15 seconds.

After 15 seconds,
If participant has not attempted/completed the task, give Level 2 prompt:
verbal reminders to use the device and verbal instructions on how to use the device,
“[Participant’s name], please read and tell me any word on the paper in front of you.”
Administrator 2 starts stop-watch at the end of verbal instruction for 15 seconds.

After 15 seconds,
If participant has not attempted/completed the task, give Level 3 prompt:
verbal and non-verbal reminder to use the device (e.g., adjusting the participant's head/finger to aim/point at the object in question),
“[Participant’s name], please read and tell me any word on the paper in front of you.”
Administrator 2 starts stop-watch at the end of verbal instruction for 15 seconds.

After 15 seconds,
If participant declares being unable to do, then score as not completed and the duration as the maximum 180 seconds, and move to Item #1b. If participant appears still to be attempting to complete the task, Administrator 1 may elect to help the participant to complete the task though completion will not be scored.

Administrator 1 signals the score to Administrator 2 for confirmation.

Item #1b Identify the correct subject (asked by Administrator 1) of two school-related documents presented.

Administrator 1 signals test item number/letter and establishes eye contact with Administrator 2.

Administrator 1 verbalizes:
“For the next activity, I am going to give you 2 pieces of paper and ask you to tell me which one is about the subject XXXXX.”

Administrator 1 places school document #1 in front of participant.
Administrator 1 verbalizes:
“I just put paper #1 in front of you. I will put your hand where the paper is. Please read it to yourself.”
Administrator 1 counts by hand/silently at the end of verbal instruction for 10 seconds.

After 10 seconds, Administrator 1 places school document #2 in front of participant.
Administrator 1 verbalizes:
“Now, I put paper #2 in front of you. I will put your hand where the paper is. Please find out for yourself what it is about.”
Administrator 1 counts by hand/silently at the end of verbal instruction for 10 seconds.

After 10 seconds, Administrator 1 verbalizes:
“Please tell me which paper, #1 or #2, is about the subject XXXXXX.”

Administrator 2 starts stop-watch at the end of initial round of instructions for scoring purpose (i.e., only stop when activity has been completed or ended).

If participant has not attempted/completed the task
Give Level 1 prompt:
"[Participant's name], use the OrCam on your eyeglasses like you did in practice. I am going to give you 2 pieces of papers and ask you to tell me which one is about the subject XXXXX."
Administrator 1 places paper #1 in front of participant.
"I just put paper #1 in front of you. I will put your hand where the paper is."
Administrator 1 guides participant's hand to the label.
"Please read it to yourself."
Administrator 1 counts by hand/silently at the end of verbal instruction for 10 seconds.

After 10 seconds, Administrator 1 places paper #2 in front of participant.
Administrator 1 verbalizes:
"Now, I just put paper #2 in front of you. I will put your hand where the paper is."
Administrator 1 guides participant's hand to the label.
"Please it to yourself."
Administrator 1 counts by hand/silently at the end of verbal instruction for 10 seconds.

After 10 seconds, Administrator 1 verbalizes:
"Please tell me which paper, #1 or #2, is about the subject XXXXXX."

If participant has not attempted/completed the task
Give Level 2 prompt:
verbal reminders to use the device and verbal instructions on how to use the device,
round of task instructions exactly as in Level 1 prompt

If participant has not attempted/completed the task
Give Level 3 prompt:
verbal and non-verbal reminder to use the device (e.g., adjusting the participant's head/finger to aim/point at the object in question),
round of task instructions exactly as in Level 1 prompt

If participant declares being unable to do, then score as not completed and the duration as the maximum 180 seconds, and move to Item #2a. If participant appears still to be attempting to complete the task, Administrator 1 may elect to help the participant to complete the task though completion will not be scored.

Administrator 1 signals the score to Administrator 2 for confirmation.

Item #2a Recognize options from a printed Menu.

Administrator 1 signals test item number/letter and establishes eye contact with Administrator 2.

Administrator 1 places the menu in front of participant. Administrator 1 checks that blue light on OrCam is on. If not, Administrator 1 taps/asks participant to tap OrCam once to wake up the OrCam.
Administrator 1 verbalizes:

"For the next activity, I just put a menu in front of you. I will put your hand where the menu is. Please read and tell me any word on the menu."

Administrator 1 counts by hand/silently at the end of verbal instruction for 10 seconds and stop the OrCam from reading (if needed). Verbalizes: "Please tell me any word on the menu."

Administrator 2 starts stop-watch at the end of verbal instruction for scoring purpose (i.e., only stop when activity has been completed or ended).

If participant has not attempted/completed the task, give Level 1 prompt:

"[Participant's name], use the OrCam on your eyeglasses like you did in practice. Please read and tell me any word on the menu." Administrator 2 starts stop-watch at the end of verbal instruction for 15 seconds.

After 15 seconds,

If participant has not attempted/completed the task, give Level 2 prompt:

verbal reminders to use the device and verbal instructions on how to use the device, "[Participant's name], please read and tell me any word on the menu." Administrator 2 starts stop-watch at the end of verbal instruction for 15 seconds.

After 15 seconds,

If participant has not attempted/completed the task, give Level 3 prompt:

verbal and non-verbal reminder to use the device (e.g., adjusting the participant's head/finger to aim/point at the object in question), "[Participant's name], please read and tell me any word on the menu." Administrator 2 starts stop-watch at the end of verbal instruction for 15 seconds.

After 15 seconds,

If participant declares being unable to do, then score as not completed and the duration as the maximum 180 seconds, and move to Item #2b. If participant appears still to be attempting to complete the task, Administrator 1 may elect to help the participant to complete the task though completion will not be scored.

Administrator 1 signals the score to Administrator 2 for confirmation.

Item #2b Select food item from a menu.

Administrator 1 signals test item number/letter and establishes eye contact with Administrator 2.

Administrator 1 uses the menu from #2a.
Administrator 1 verbalizes:

"Now, please tell me what you would want to order from the menu."

Administrator 1 counts by hand/silently at the end of verbal instruction for 10 seconds and stop the OrCam from reading (if needed). Verbalizes: "Please tell me what you would want to order from the menu."

Administrator 2 starts stop-watch at the end of verbal instruction for scoring purpose (i.e., only stop when activity has been completed or ended).

If participant has not attempted/completed the task, give Level 1 prompt:

"[Participant's name], use the OrCam on your eyeglasses like you did in practice. Please tell me what you would want to order from the menu." Administrator 2 starts stop-watch at the end of verbal instruction for 15 seconds.

After 15 seconds,
If participant has not attempted/completed the task, give Level 2 prompt:
verbal reminders to use the device and verbal instructions on how to use the device,
"[Participant's name], please tell me what you would want to order from the menu."
Administrator 2 starts stop-watch at the end of verbal instruction for 15 seconds.

After 15 seconds,
If participant has not attempted/completed the task, give Level 3 prompt:
verbal and non-verbal reminder to use the device (e.g., adjusting the participant's head/finger to aim/point at the object in question),
"[Participant's name], please tell me what you would want to order from the menu."
Administrator 2 starts stop-watch at the end of verbal instruction for 15 seconds.

After 15 seconds,
If participant declares being unable to do, then score as not completed and the duration as the maximum 180 seconds, and move to Item #5a. If participant appears still to be attempting to complete the task, Administrator 1 may elect to help the participant to complete the task though completion will not be scored.

Administrator 1 signals the score to Administrator 2 for confirmation.

Item #5a Recognize text on a page in a book.

Administrator 1 signals test item number/letter and establishes eye contact with Administrator 2.

Administrator 1 places a children's book (preferably without pictures), opened to a page (the content of which is familiar to the Administrator 1) in front of participant. Administrator 1 checks that blue light on OrCam is on. If not, Administrator 1 taps/asks participant to tap OrCam once to wake up the OrCam.
Administrator 1 verbalizes:
"For the next activity, I just put a book page in front of you. I will put your hand where the page is. Please read and tell me any word on the page."
Administrator 1 counts by hand/silently at the end of verbal instruction for 10 seconds and stop the OrCam from reading (if needed). Verbalizes: "Please tell me any word on the page."

Administrator 2 starts stop-watch at the end of verbal instruction for scoring purpose (i.e., only stop when activity has been completed or ended).

If participant has not attempted/completed the task, give Level 1 prompt:
"[Participant's name], use the OrCam on your eyeglasses like you did in practice. Please read and tell me any word on the page." Administrator 2 starts stop-watch at the end of verbal instruction for 15 seconds.

After 15 seconds,
If participant has not attempted/completed the task, give Level 2 prompt:

verbal reminders to use the device and verbal instructions on how to use the device, "[Participant's name], please read and tell me any word on the page." Administrator 2 starts stop-watch at the end of verbal instruction for 15 seconds.

After 15 seconds,

If participant has not attempted/completed the task, give Level 3 prompt:

verbal and non-verbal reminder to use the device (e.g., adjusting the participant's head/finger to aim/point at the object in question),

"[Participant's name], please read and tell me any word on the page." Administrator 2 starts stop-watch at the end of verbal instruction for 15 seconds.

After 15 seconds,

If participant declares being unable to do, then score as not completed and the duration as the maximum 180 seconds, and move to Item #5b. If participant appears still to be attempting to complete the task, Administrator 1 may elect to help the participant to complete the task though completion will not be scored.

Administrator 1 signals the score to Administrator 2 for confirmation.

Item #5b Answer specific question relating to content from a page of a book.

Administrator 1 signals test item number/letter and establishes eye contact with Administrator 2.

Administrator 1 places a children's book (preferably without pictures), opened to a page (the content of which is familiar to the Administrator 1) in front of participant. Administrator 1 can elect to use the same set up as in #5a.

Administrator 1 verbalizes:

"Now from this page of the book, please read and tell me the XXXXXX (e.g., what was the best friend's name? Or, what did Robbie lose?).

Administrator 1 counts by hand/silently at the end of verbal instruction for 10 seconds and stop the OrCam from reading (if needed). Verbalizes: "Please tell me the XXXXXX (e.g., what was the best friend's name? Or, what did Robbie lose?)."

Administrator 2 starts stop-watch at the end of verbal instruction for scoring purpose (i.e., only stop when activity has been completed or ended).

If participant has not attempted/completed the task, give Level 1 prompt:

"[Participant's name], use the OrCam on your eyeglasses like you did in practice. Please read and tell me the XXXXXX (e.g., what was the best friend's name? Or, what did Robbie lose?)."

Administrator 2 starts stop-watch at the end of verbal instruction for 15 seconds.

After 15 seconds,

If participant has not attempted/completed the task, give Level 2 prompt:

verbal reminders to use the device and verbal instructions on how to use the device, "[Participant's name], please read and tell me the XXXXXX (e.g., what was the best friend's name? Or, what did Robbie lose?)." Administrator 2 starts stop-watch at the end of verbal instruction for 15 seconds.

After 15 seconds,

If participant has not attempted/completed the task, give Level 3 prompt:

verbal and non-verbal reminder to use the device (e.g., adjusting the participant's head/finger to aim/point at the object in question),

"[Participant's name], please read and tell me the XXXXXX (e.g., what was the best friend's name? Or, what did Robbie lose?)." Administrator 2 starts stop-watch at the end of verbal instruction for 15 seconds.

After 15 seconds,

If participant declares being unable to do, then score as not completed and the duration as the maximum 180 seconds, and move to Item #6a. If participant appears still to be attempting to complete the task, Administrator 1 may elect to help the participant to complete the task though completion will not be scored.

Administrator 1 signals the score to Administrator 2 for confirmation.

Item #6a Recognize the color of a uniformed, single, primary colored item.

Administrator 1 signals test item number/letter and establishes eye contact with Administrator 2.

Administrator 1 places a piece (~10x10 cm or larger) of paper of a primary color (red, yellow, or blue) in front of participant. Administrator 1 checks that blue light on OrCam is on. If not, Administrator 1 taps/asks participant to tap OrCam once to wake up the OrCam.
Administrator 1 verbalizes:

"For this next activity, I just put a piece of paper in front of you. I will put your hand where the paper is. Please tell me the color of the piece of paper."

Administrator 2 starts stop-watch at the end of verbal instruction for 15 seconds.

Administrator 2 starts stop-watch at the end of verbal instruction for scoring purpose (i.e., only stop when activity has been completed or ended).

If participant has not attempted/completed the task, give Level 1 prompt:

"[Participant's name], use the OrCam on your eyeglasses like you did in practice. Please tell me the color of the piece of paper in front of you." Administrator 2 starts stop-watch at the end of verbal instruction for 15 seconds.

After 15 seconds,

If participant has not attempted/completed the task, give Level 2 prompt:

verbal reminders to use the device and verbal instructions on how to use the device,
"[Participant's name], please tell me the color of the piece of paper in front of you."
Administrator 2 starts stop-watch at the end of verbal instruction for 15 seconds.

After 15 seconds,

If participant has not attempted/completed the task, give Level 3 prompt:

verbal and non-verbal reminder to use the device (e.g., adjusting the participant's head/finger to aim/point at the object in question),
"[Participant's name], please tell me the color of the piece of paper in front of you."
Administrator 2 starts stop-watch at the end of verbal instruction for 15 seconds.

After 15 seconds,

If participant declares being unable to do, then score as not completed and the duration as the maximum 180 seconds, and move to Item #6b. If participant appears still to be attempting to complete the task, Administrator 1 may elect to help the participant to complete the task though completion will not be scored.

Administrator 1 signals the score to Administrator 2 for confirmation.

Item #6b Recognize one of two colored items.

Administrator 1 signals test item number/letter and establishes eye contact with Administrator 2.

Administrator 1 verbalizes:

"For the next activity, I am going to give you 2 pieces of papers and ask you to tell me which one has the color XXXXXX."

Administrator 1 places colored paper #1 in front of participant.

"I just put colored paper #1 in front of you. I will put your hand where the paper is. Please find out for yourself what color it is."

Administrator 1 counts by hand/silently at the end of verbal instruction for 10 seconds.

After 10 seconds, Administrator 1 places colored paper #2 in front of participant.

Administrator 1 verbalizes:

"Now I just put colored paper #2 in front of you. I will put your hand where the paper is. Please find out for yourself what color it is."

Administrator 1 counts by hand/silently at the end of verbal instruction for 10 seconds.

After 10 seconds, Administrator 1 verbalizes:

"Please tell me which paper, #1 or #2, has the color XXXXXX."

Administrator 2 starts stop-watch at the end of initial round of instructions for scoring purpose (i.e., only stop when activity has been completed or ended).

If participant has not attempted/completed the task

Give Level 1 prompt:

"[Participant's name], use the OrCam on your eyeglasses like you did in practice. I am going to give you 2 pieces of papers and ask you to tell me which one has the color XXXXXX."

Administrator 1 places paper #1 in front of participant.

"I just put colored paper #1 in front of you. I will put your hand where the paper is. Please find out for yourself what color it is."

Administrator 1 counts by hand/silently at the end of verbal instruction for 10 seconds.

After 10 seconds, Administrator 1 places paper #2 in front of participant.

Administrator 1 verbalizes:

"Now I just put colored paper #2 in front of you. I will put your hand where the paper is. Please find out for yourself what color it is."

Administrator 1 counts by hand/silently at the end of verbal instruction for 10 seconds.

After 10 seconds, Administrator 1 verbalizes:

“Please tell me which paper, #1 or #2, has the color XXXXXX.”

If participant has not attempted/completed the task
Give Level 2 prompt:
verbal reminders to use the device and verbal instructions on how to use the device,
round of task instructions exactly as in Level 1 prompt

If participant has not attempted/completed the task
Give Level 3 prompt:
verbal and non-verbal reminder to use the device (e.g., adjusting the participant's head/finger to aim/point at the object in question),
round of task instructions exactly as in Level 1 prompt

If participant declares being unable to do, then score as not completed and the duration as the maximum 180 seconds, and move to Item #3a. If participant appears still to be attempting to complete the task, Administrator 1 may elect to help the participant to complete the task though completion will not be scored.

Administrator 1 signals the score to Administrator 2 for confirmation.

Item #3a Recognize a room sign.

Administrator 1 signals test item number/letter and establishes eye contact with Administrator 2.

Administrator 1 verbalizes:
“For the next activity, we are going to go outside of the room. I am going to ask you about signs.”
Administrator 1 positions participant in front of a room sign (e.g., room number, bathroom sign), within 2 feet of the sign. Administrator 1 checks that blue light on OrCam is on. If not, Administrator 1 taps/asks participant to tap OrCam once to wake up the OrCam. Verbalizes:
“You are standing in front of a sign for the room. I will put your hand where the sign is. Please read and tell me what the sign says.”
Administrator 2 starts stop-watch at the end of verbal instruction for 15 seconds.

Administrator 2 starts stop-watch at the end of verbal instruction for scoring purpose (i.e., only stop when activity has been completed or ended).

If participant has not attempted/completed the task, give Level 1 prompt:
“[Participant’s name], use the OrCam on your eyeglasses like you did in practice. Please read and tell me what the sign says.” Administrator 2 starts stop-watch at the end of verbal instruction for 15 seconds.

After 15 seconds,
If participant has not attempted/completed the task, give Level 2 prompt:
verbal reminders to use the device and verbal instructions on how to use the device,
“[Participant’s name], please read and tell me what the sign says.” Administrator 2 starts stop-watch at the end of verbal instruction for 15 seconds.

After 15 seconds,

If participant has not attempted/completed the task, give Level 3 prompt:
verbal and non-verbal reminder to use the device (e.g., adjusting the participant's head/finger to aim/point at the object in question),
"[Participant's name], please read and tell me what the sign says." Administrator 2 starts stop-watch at the end of verbal instruction for 15 seconds.

After 15 seconds,
If participant declares being unable to do, then score as not completed and the duration as the maximum 180 seconds, and move to Item #3b. If participant appears still to be attempting to complete the task, Administrator 1 may elect to help the participant to complete the task though completion will not be scored.

Administrator 1 signals the score to Administrator 2 for confirmation.

Item #3b Identify which room to enter based on need/preference.

Administrator 1 signals test item number/letter and establishes eye contact with Administrator 2.

Administrator 1 verbalizes:
"For the next activity, I am going to ask you to choose between two room signs."
Administrator 1 places participant in front of sign #1, within 2 feet of the sign.
"You are in front of room sign #1. I will put your hand where the sign is. Please read to yourself what the sign says."
Administrator 1 counts by hand/silently at the end of verbal instruction for 10 seconds.

After 10 seconds, Administrator 1 places participant in front of sign #2, within 2 feet of the sign.
Administrator 1 verbalizes:
"You are in front of room sign #2. I will put your hand where the sign is. Please read to yourself what the sign says."
Administrator 1 counts by hand/silently at the end of verbal instruction for 10 seconds.

After 10 seconds, Administrator 1 verbalizes:
"Please tell me which room, #1 or #2, you would go in to 'XXXXX' (e.g., cook food, use the restroom, etc.)"

Administrator 2 starts stop-watch at the end of initial round of instructions for scoring purpose (i.e., only stop when activity has been completed or ended).

If participant has not attempted/completed the task
Give Level 1 prompt:
"[Participant's name], use the OrCam on your eyeglasses like you did in practice. I am going to ask you to choose between two room signs."
Administrator 1 places participant in front of sign #1, within 2 feet of the sign.
"You are in front of room sign #1. I will put your hand where the sign is. Please read to yourself what the sign says."
Administrator 1 counts by hand/silently at the end of verbal instruction for 10 seconds.

After 10 seconds, Administrator 1 places participant in front of sign #2, within 2 feet of the sign.

Administrator 1 verbalizes:
"You are in front of room sign #2. I will put your hand where the sign is. Please read to yourself what the sign says."
Administrator 1 counts by hand/silently at the end of verbal instruction for 10 seconds.

After 10 seconds, Administrator 1 verbalizes:
"Please tell us which room, #1 or #2, you would go in to 'XXXXX' (e.g., cook food, use the restroom, etc.)"

If participant has not attempted/completed the task
Give Level 2 prompt:
verbal reminders to use the device and verbal instructions on how to use the device,
round of task instructions exactly as in Level 1 prompt

If participant has not attempted/completed the task
Give Level 3 prompt:
verbal and non-verbal reminder to use the device (e.g., adjusting the participant's head/finger to aim/point at the object in question),
round of task instructions exactly as in Level 1 prompt

If participant declares being unable to do, then score as not completed and the duration as the maximum 180 seconds, and move to Item #7a. If participant appears still to be attempting to complete the task, Administrator 1 may elect to help the participant to complete the task though completion will not be scored.

Administrator 1 signals the score to Administrator 2 for confirmation.

Item #7a Recognize a facing person with prompting.

Administrator 1 signals test item number/letter and establishes eye contact with Administrator 2.

A study team member, different from the Administrator 1, positions (~6 ft) in front of participant before verbal instructions provided by the Administrator 1. Administrator 1 moves out of participant's field of vision.

Administrator 1 checks that blue light on OrCam is on. If not, Administrator 1 taps/asks participant to tap OrCam once to wake up the OrCam. Verbalizes:
"For the next activity, I am going to ask you about the person in front of you. Please tell me who that person is."
Administrator 2 starts stop-watch at the end of verbal instruction for 15 seconds.

Administrator 2 starts stop-watch at the end of verbal instruction for scoring purpose (i.e., only stop when activity has been completed or ended).

After 15 seconds,
If participant has not attempted/completed the task, give Level 1 prompt:
"[Participant's name], use the OrCam on your eyeglasses like you did in practice. Please tell me who is the person in front of you." Administrator 2 starts stop-watch at the end of verbal instruction for 15 seconds.

After 15 seconds,
If participant has not attempted/completed the task, give Level 2 prompt:
verbal reminders to use the device and verbal instructions on how to use the device, "[Participant's name], please tell me who is the person in front of you." Administrator 2 starts stop-watch at the end of verbal instruction for 15 seconds.

After 15 seconds,
If participant has not attempted/completed the task, give Level 3 prompt:
verbal and non-verbal reminder to use the device (e.g., adjusting the participant's head/finger to aim/point at the object in question),
"[Participant's name], please tell me tell me who is the person in front of you." Administrator 2 starts stop-watch at the end of verbal instruction for 15 seconds.

After 15 seconds,
If participant declares being unable to do, then score as not completed and the duration as the maximum 180 seconds, and move to Item #7b. If participant appears still to be attempting to complete the task, Administrator 1 may elect to help the participant to complete the task though completion will not be scored.

Administrator 1 signals the score to Administrator 2 for confirmation.

Item #7b Recognize a facing person without prompt.

Administrator 1 signals test item number/letter and establishes eye contact with Administrator 2.

A study team member, different from the Administrator 1, positions (~6 ft) in front of participant before, during, or after verbal instructions given by the Administrator 1. Administrator 1 moves out of participant's field of vision.
Verbal instruction to be provided by the Administrator 1:
"In the next few minutes, there will be another person in front of you. Please tell me who that person is whenever you see them."
Administrator 2 starts stop-watch at the end of verbal instruction for 15 seconds.

Administrator 2 starts stop-watch at the end of verbal instruction for scoring purpose (i.e., only stop when activity has been completed or ended).

After 15 seconds,
If participant has not attempted/completed the task, give Level 1 prompt:
"[Participant's name], use the OrCam on your eyeglasses like you did in practice. In the next few minutes, there will be a person in front of you. Please tell me who that person is whenever you see them." Administrator 2 starts stop-watch at the end of verbal instruction for 15 seconds.

After 15 seconds,
If participant has not attempted/completed the task, give Level 2 prompt:
verbal reminders to use the device and verbal instructions on how to use the device,

“[Participant’s name], in the next few minutes, there will be another person in front of you. Please tell me who that person is whenever you see them.” Administrator 2 starts stop-watch at the end of verbal instruction for 15 seconds.

After 15 seconds,
If participant has not attempted/completed the task, give Level 3 prompt:
verbal and non-verbal reminder to use the device (e.g., adjusting the participant's head/finger to aim/point at the object in question),
“[Participant’s name], in the next few minutes, there will be another person in front of you. Please tell me who that person is whenever you see them.” Administrator 2 starts stop-watch at the end of verbal instruction for 15 seconds.

After 15 seconds,
If participant declares being unable to do, then score as not completed and the duration as the maximum 180 seconds, and move to Item #8a. If participant appears still to be attempting to complete the task, Administrator 1 may elect to help the participant to complete the task though completion will not be scored.

If **proceeding** with exploratory items, Administrator 1 verbalizes:
“Let’s go back to the room.”
If **not proceeding** with exploratory items (e.g., participant is fatigued or inattentive, or if time is running out), Administrator 1 verbalizes:
“This is the end of these activities. Thank you for working with me. Let’s go back to the room.”

Administrator 1 signals the score to Administrator 2 for confirmation.

Item #8a Report the day and date.

Administrator 1 signals test item number/letter and establishes eye contact with Administrator 2.

Administrator 1 checks that blue light on OrCam is on. If not, Administrator 1 taps/asks participant to tap OrCam once to wake up the OrCam. Verbalizes:
“Please tell me today’s date.”
Administrator 2 starts stop-watch at the end of verbal instruction for 15 seconds.

Administrator 2 starts stop-watch at the end of verbal instruction for scoring purpose (i.e., only stop when activity has been completed or ended).

After 15 seconds,
If participant has not attempted/completed the task, give Level 1 prompt:
“[Participant’s name], use the OrCam on your eyeglasses like you did in practice. Please tell me today’s date.” Administrator 2 starts stop-watch at the end of verbal instruction for 15 seconds.

After 15 seconds,
If participant has not attempted/completed the task, give Level 2 prompt:
verbal reminders to use the device and verbal instructions on how to use the device,
“[Participant’s name], please tell me today’s date.” Administrator 2 starts stop-watch at the end of verbal instruction for 15 seconds.

After 15 seconds,

If participant has not attempted/completed the task, give Level 3 prompt:

verbal and non-verbal reminder to use the device (e.g., adjusting the participant's head/finger to aim/point at the object in question),

“[Participant’s name], please tell me today’s date.” Administrator 2 starts stop-watch at the end of verbal instruction for 15 seconds.

After 15 seconds,

If participant declares being unable to do, then score as not completed and the duration as the maximum 180 seconds, and move to Item #8b. If participant appears still to be attempting to complete the task, Administrator 1 may elect to help the participant to complete the task though completion will not be scored.

Administrator 1 signals the score to Administrator 2 for confirmation.

Item #8b Identify the day and answer test admin’s question (how many days until Sunday)

Administrator 1 signals test item number/letter and establishes eye contact with Administrator 2.

Administrator 1 verbalizes:

“Please tell me what day of the week is today, and how many days until Sunday.”

Administrator 2 starts stop-watch at the end of verbal instruction for 15 seconds.

Administrator 2 starts stop-watch at the end of verbal instruction for scoring purpose (i.e., only stop when activity has been completed or ended).

After 15 seconds,

If participant has not attempted/completed the task, give Level 1 prompt:

“[Participant’s name], use the OrCam on your eyeglasses like you did in practice. Please tell me what day of the week is today, and how many days until Sunday.” Administrator 2 starts stop-watch at the end of verbal instruction for 15 seconds.

After 15 seconds,

If participant has not attempted/completed the task, give Level 2 prompt:

verbal reminders to use the device and verbal instructions on how to use the device,

“[Participant’s name], please tell me what day of the week is today, and how many days until Sunday.” Administrator 2 starts stop-watch at the end of verbal instruction for 15 seconds.

After 15 seconds,

If participant has not attempted/completed the task, give Level 3 prompt:

verbal and non-verbal reminder to use the device (e.g., adjusting the participant's head/finger to aim/point at the object in question),

“[Participant’s name], please tell me what day of the week is today, and how many days until Sunday.” Administrator 2 starts stop-watch at the end of verbal instruction for 15 seconds.

After 15 seconds,

If participant declares being unable to do, then score as not completed and the duration as the maximum 180 seconds, and move to Item #9a. If participant appears still to be

attempting to complete the task, Administrator 1 may elect to help the participant to complete the task though completion will not be scored.

Administrator 1 signals the score to Administrator 2 for confirmation.

Item #9a Recognize a snack product by its label.

Administrator 1 signals test item number/letter and establishes eye contact with Administrator 2.

Administrator 1 places a bag of snack (e.g., Goldfish crackers, Teddy Grahams, pretzels) in front of study participant. Administrator 1 checks that blue light on OrCam is on. If not, Administrator 1 taps/asks participant to tap OrCam once to wake up the OrCam. Verbalizes:
"For the next activity, I am going to ask you about snack labels. I will put or hold a snack bag/container in front of you. Please read and tell me the name of the snack bag/container."
Administrator 2 starts stop-watch at the end of verbal instruction for 15 seconds.

Administrator 2 starts stop-watch at the end of verbal instruction for scoring purpose (i.e., only stop when activity has been completed or ended).

After 15 seconds,
If participant has not attempted/completed the task, give Level 1 prompt:
"[Participant's name], use the OrCam on your eyeglasses like you did in practice. I will put or hold a snack bag/container in front of you. Please read and tell me the name of the snack bag/container." Administrator 2 starts stop-watch at the end of verbal instruction for 15 seconds.

After 15 seconds,
If participant has not attempted/completed the task, give Level 2 prompt:
verbal reminders to use the device and verbal instructions on how to use the device,
"[Participant's name], please read and tell me the name of the snack bag/container in front of you." Administrator 2 starts stop-watch at the end of verbal instruction for 15 seconds.

After 15 seconds,
If participant has not attempted/completed the task, give Level 3 prompt:
verbal and non-verbal reminder to use the device (e.g., adjusting the participant's head/finger to aim/point at the object in question),
"[Participant's name], please read and tell me the name of the snack bag/container in front of you." Administrator 2 starts stop-watch at the end of verbal instruction for 15 seconds.

After 15 seconds,
If participant declares being unable to do, then score as not completed and the duration as the maximum 180 seconds, and move to Item #9b. If participant appears still to be attempting to complete the task, Administrator 1 may elect to help the participant to complete the task though completion will not be scored.

Administrator 1 signals the score to Administrator 2 for confirmation.

Item #9b Recognize and take the preferred snack option.

Administrator 1 signals test item number/letter and establishes eye contact with Administrator 2.

Administrator 1 verbalizes:
"For the next activity, I am going to ask you to choose from two snacks."
Administrator 1 places snack bag #1 (e.g. Goldfish crackers, Teddy Grahams, pretzels) in front of participant.
"Snack #1 is in front of you. Please read to yourself what it is."
Administrator 1 counts by hand/silently at the end of verbal instruction for 10 seconds.

After 10 seconds, Administrator 1 places snack #2 in front of participant.
Administrator 1 verbalizes:
"Snack #2 is in front of you. Please read to yourself what it is."
Administrator 1 counts by hand/silently at the end of verbal instruction for 10 seconds.

After 10 seconds, Administrator 1 verbalizes:
"Please tell me which snack, #1 or #2, you prefer."

Administrator 2 starts stop-watch at the end of initial round of instructions for scoring purpose (i.e., only stop when activity has been completed or ended).

If participant has not attempted/completed the task
Give Level 1 prompt:
"[Participant's name], use the OrCam on your eyeglasses like you did in practice. I am going to ask you to choose from two snacks."
Administrator 1 places snack bag #1 (e.g. Goldfish crackers, Teddy Grahams, pretzels) in front of participant.
"Snack #1 is in front of you. Please read to yourself what it is."
Administrator 1 counts by hand/silently at the end of verbal instruction for 10 seconds.

After 10 seconds, Administrator 1 places snack #2 in front of participant.
Administrator 1 verbalizes:
"Snack #2 is in front of you. Please read to yourself what it is."
Administrator 1 counts by hand/silently at the end of verbal instruction for 10 seconds.

After 10 seconds, Administrator 1 verbalizes:
"Please tell me which snack, #1 or #2, you prefer."

If participant has not attempted/completed the task
Give Level 2 prompt:
verbal reminders to use the device and verbal instructions on how to use the device, round of task instructions exactly as in Level 1 prompt

If participant has not attempted/completed the task
Give Level 3 prompt:
verbal and non-verbal reminder to use the device (e.g., adjusting the participant's head/finger to aim/point at the object in question),
round of task instructions exactly as in Level 1 prompt

If participant declares being unable to do, then score as not completed and the duration as the maximum 180 seconds, and move to Item #10a. If participant appears still to be attempting to complete the task, Administrator 1 may elect to help the participant to complete the task though completion will not be scored.

Administrator 1 signals the score to Administrator 2 for confirmation.

Item #10a Recognize options from a Menu on a screen device.

Administrator 1 signals test item number/letter and establishes eye contact with Administrator 2.

Administrator 1 displays a menu on a screen device.
Administrator 1 checks that blue light on OrCam is on. If not, Administrator 1 taps/asks participant to tap OrCam once to wake up the OrCam. Verbalizes:
"For the next activity, I am going to ask you about a list of items. I just put a list on a(n) XXXXXX (e.g., iPad, computer, etc.) in front of you. I will put your hand where the XXXX (e.g., iPad, computer, etc.) is. Please read and tell me any word on the list."
Administrator 2 starts stop-watch at the end of verbal instruction for 15 seconds.

Administrator 2 starts stop-watch at the end of verbal instruction for scoring purpose (i.e., only stop when activity has been completed or ended).

After 15 seconds,
If participant has not attempted/completed the task, give Level 1 prompt:
"[Participant's name], use the OrCam on your eyeglasses like you did in practice. Please read and tell me any word on the list." Administrator 2 starts stop-watch at the end of verbal instruction for 15 seconds.

After 15 seconds,
If participant has not attempted/completed the task, give Level 2 prompt:
verbal reminders to use the device and verbal instructions on how to use the device, "[Participant's name], please read and tell me any word on the list." Administrator 2 starts stop-watch at the end of verbal instruction for 15 seconds.

After 15 seconds,
If participant has not attempted/completed the task, give Level 3 prompt:
verbal and non-verbal reminder to use the device (e.g., adjusting the participant's head/finger to aim/point at the object in question),
"[Participant's name], please read and tell me any word on the list." Administrator 2 starts stop-watch at the end of verbal instruction for 15 seconds.

After 15 seconds,
If participant declares being unable to do, then score as not completed and the duration as the maximum 180 seconds, and move to Item #10b. If participant appears still to be attempting to complete the task, Administrator 1 may elect to help the participant to complete the task though completion will not be scored.

Administrator 1 signals the score to Administrator 2 for confirmation.

Item #10b Recognize options from a Menu list and decide whether an action can be done.

Administrator 1 signals test item number/letter and establishes eye contact with Administrator 2.

Administrator 1 displays a list on a screen device. Administrator 1 may elect to use the same set-up in #10a.
Administrator 1 verbalizes:
"For the next activity, I am going to ask you whether an item is on the list. Please read and tell me whether you can XXXXXX (e.g., play song XXXXXX? Or, do XXXXXX?)."
Administrator 2 starts stop-watch at the end of verbal instruction for 15 seconds.

Administrator 2 starts stop-watch at the end of verbal instruction for scoring purpose (i.e., only stop when activity has been completed or ended).

After 15 seconds,
If participant has not attempted/completed the task, give Level 1 prompt:
"[Participant's name], use the OrCam on your eyeglasses like you did in practice. Please read and tell me whether you can XXXXXX (e.g., play song XXXXXX? Or, do XXXXXX?)."
Administrator 2 starts stop-watch at the end of verbal instruction for 15 seconds.

After 15 seconds,
If participant has not attempted/completed the task, give Level 2 prompt:
verbal reminders to use the device and verbal instructions on how to use the device,
"[Participant's name], please read and tell me whether you can XXXXXX (e.g., play song XXXXXX? Or, do XXXXXX?)." Administrator 2 starts stop-watch at the end of verbal instruction for 15 seconds.

After 15 seconds,
If participant has not attempted/completed the task, give Level 3 prompt:
verbal and non-verbal reminder to use the device (e.g., adjusting the participant's head/finger to aim/point at the object in question),
"[Participant's name], please read and tell me whether you can XXXXXX (e.g., play song XXXXXX? Or, do XXXXXX?)." Administrator 2 starts stop-watch at the end of verbal instruction for 15 seconds.

After 15 seconds,
If participant declares being unable to do, then score as not completed and the duration as the maximum 180 seconds and end the Function Test. If participant appears still to be attempting to complete the task, Administrator 1 may elect to help the participant to complete the task though completion will not be scored.

Administrator 1 signals the score to Administrator 2 for confirmation.

Administrator 1 verbalizes:
"This is the end of these activities. Thank you for working with me."
