# Supplementary material for: Prospective pilot safety, feasibility study of an optic-to-audio device for children with CLN3 disease
Source: Orphanet J Rare Dis. 2026 Apr 3;21:199. doi: 10.1186/s13023-026-04319-0 (PMC13173725; doi:10.1186/s13023-026-04319-0)
Supplement: Supplementary file 3 — Supplementary Material 3: Additional File 3. OrCam training manual. [file 13023_2026_4319_MOESM3_ESM.pdf]

ADDITIONAL FILE 3

OrCam Training Manual

OUTLINE

Parents & Child (OrCam #1)
***This will be done during training sessions involving both the participant and the parent(s). This will typically be during study visit Day 1 afternoon, Day 2, Day 5.

- I. **Introduction to OrCam & sounds**
- II. **Pairing OrCam w/ glasses**
  (Order of presentation and number of repeats optional, based on trainer's assessment of participant's needs.)
- III. **Reading labels (tap)**
- IV. **Reading school documents (tap)**
- V. **Reading pages in a book (tap)**

Break & Transition

- I. **Reading signs (tap)**
- II. **Identifying face(s) (tap)**

Transition

- I. **Identifying color(s) (hold finger)**

Break

***If the below items were evaluated during baseline, then training will need to be done for re-evaluation at 1 week/1 month.
If the below items were not evaluated during baseline because of time/cooperation issues, then training does not need to be done. These functions can be mentioned to parents so they may choose to explore on their own.***

- I. **Reading iPad/screen**
- II. **Reading snack labels**
- III. **Finding out time and date**

Parents (OrCam #2)
***This will be done during training session with the parent(s), while the participant undergoes baseline Feasibility and Function Tests. This will be during study visit Day 1.

- I. **Viewing video tutorials ppt**
- II. **Positioning of object(s)**
- III. **Pairing OrCam to phone**
- IV. **Pairing face(s) to name(s)**

INTRODUCTION

"Now, I am going to teach you how to use the OrCam to do several things. We can take breaks whenever you need them, just let me know."

If participants appear comfortable and familiar with the eyeglass/OrCam, may elect to do the next section at the end of training session, and move to "Introduction to Sounds" section.

Attaching the OrCam to the Glasses

"Let me show you the OrCam and the glasses. The OrCam sticks to the side of the glasses, near your ear. It has a camera, a speaker, and will speak into your ear. I will teach you how to use the OrCam for some activities. Some of the people on the team may come in and out to help with the training. Before we start, do you have any question? Okay, let's begin."

Trainer places the glasses in front of participant and verbalizes:
"I just put a pair of glasses in front of you. Go ahead and feel them. On one side of the glasses, you will feel a bump. You want to put the bump to your right-hand side. That is where the OrCam will attach to the glasses."

After 10 seconds, trainer places the OrCam device in front of participant and verbalizes:
"Please put the glasses down. I just put the OrCam in front of you. Go ahead and feel it."

After 10 seconds, trainer verbalizes:
"Please put the OrCam down. Now, I will show you how to attach the OrCam to the glasses. To do this, I will touch and move your hands. Let me know if any of this makes you uncomfortable."
Trainer positions the OrCam with the metal end pointing towards and the touch bar facing to the right of the participant.

Trainer verbalizes and correspondingly moves the participant's hand:
"I will move your hand to feel the pointy end of the OrCam. You want to put the pointy end towards you. Now, I will move your hand to feel the raised line on the side of the OrCam. You want to put the raised line on your right-hand side.
Now let's bring the OrCam close to the glasses. The OrCam will attach to the bump on the glasses like this. You will hear a snap when they attach."

"Let's practice this. The glasses and OrCam are in front of you."

*** Prompt through the steps, adjust them as needed ***

- "Put the OrCam with the pointy end towards you, and the raised line facing outside"
- "Find the bump on the glasses"
- "Bring them together to attach them."
- "The snap means they are attached"

** repeat until participant is able to do task independently, maximum 3 repeats **

Introduction to Sounds
"The OrCam makes different sounds to let you know what it is doing. I will go over these sounds with you now."

Play sound
"This is the click." *Play sound*. "Did you hear that? When you hear this click, keep your head still and look straight ahead. The click means the OrCam is starting to do what you wanted it to do."

Play sound
"This is the camera sound." *Play sound.* "Did you hear that? When you hear the camera sound, keep your head still and look straight ahead. The camera means the OrCam is taking a picture of what you wanted it to see. After the camera sound, you can relax."

Play sound
"This is the chime." *Play sound.* "Did you hear that? When you hear the chime, you can move on to the next task. The chime means the OrCam has finished doing what you wanted it to do."

READING

"Let's put on the glasses and OrCam. Now, you are ready to learn how to use the OrCam to read words. Remember to keep your head still and aim the OrCam at what you would like to read."

Labels
"We will first practice reading a label using the OrCam. I will guide your hand and finger to show you. To read, first feel with your hand where the item is. Put your head so that you are looking straight at the item. Take your hand away from the item. Tap your pointer finger ONCE on the raised line on the OrCam." *Guide trainee's hand and finger during instructions.*

"After tapping, you keep your head still and wait. The OrCam will go through the sounds you heard before. Then it will read or speak into your ear, so listen carefully."

"It is very important to face what you would like to read, to hold your head still, to tap the OrCam and to listen. Let's practice this!"

***Prompt with practice labels, adjust them as needed* **

- "Please read and tell me what the label in front of you says."
  (***Trainer may need to tap once prior to wake up the OrCam.***)
- (Chime sound)→ "After you hear the chime, you can relax."
- "We will try this again."

** repeat until participant is able to do task independently, maximum 3 repeats **

School Documents

"We will now practice reading a school paper using the OrCam. Like you did before, to read, first feel with your hand where the item is. Put your head so that you are looking straight at the item. Take your hand away from the item. Tap your pointer finger ONCE on the raised line on the OrCam." *Guide trainee's hand and finger during instructions.*

"After tapping, you keep your head still and wait. The OrCam will go through the sounds you heard before. Then it will read or speak into your ear, so listen carefully."

"It is very important to face what you would like to read, to hold your head still, to tap the OrCam and to listen. Let's practice this!"

***Prompt with practice document, adjust them as needed ***

- "Please read the school paper in front of you and tell me what it says."
- (Chime sound)→ "After you hear the chime, you can relax."
- "We will try this again."

** repeat until participant is able to do task independently, maximum 3 repeats **

Menu

"We will now practice reading a menu using the OrCam. Like you did before, to read, first feel with your hand where the item is. Put your head so that you are looking straight at the item. Take your hand away from the item. Tap your pointer finger ONCE on the raised line on the OrCam." *Guide trainee's hand and finger during instructions.*

"After tapping, you keep your head still and wait. The OrCam will go through the sounds you heard before. Then it will read or speak into your ear, so listen carefully."

"It is very important to face what you would like to read, to hold your head still, to tap the OrCam and to listen. Let's practice this!"

***Prompt with practice document, adjust them as needed ***

- "Please read the menu in front of you and tell me what it says."
- (Chime sound)→ "After you hear the chime, you can relax."
- "We will try this again."

** repeat until participant is able to do task independently, maximum 3 repeats **

Book

"We will now practice reading a page in a book using the OrCam. Like you did before, to read, first feel with your hand where the item is. Put your head so that you are looking straight at the item. Take your hand away from the item. Tap your pointer finger ONCE on the raised line on the OrCam." *Guide trainee's hand and finger during instructions.*

"After tapping, you keep your head still and wait. The OrCam will go through the sounds you heard before. Then it will read or speak into your ear, so listen carefully."

"It is <u>very</u> important to face what you would like to read, to hold your head still, to tap the OrCam and to listen. Let's practice this!"

**Prompt with page in book, adjust them as needed **

- "Please read the book page in front of you and tell me what it says."
- (Chime sound)→ "After you hear the chime, you can relax."
- "We will try this again."

** repeat until participant is able to do task independently, maximum 3 repeats **

STOP GESTURE

"Now, I will show you how to stop the OrCam from reading. To stop the OrCam, you put your hand out in front of you like you are telling someone to stop or to give a high five. Can you show me what that looks like?" *Guide trainee's hand to be like a stop sign.* "First, please use the OrCam to read the book page in front of you. Then, I will ask you to stop the OrCam."

Prompt with a page of a book, adjust them as needed

- "Please read the book page in front of you."
- "Now place your hand like you are telling someone to stop or like a high five."
- "You just stopped it from reading"
- "Let's try that again."

** repeat until participant is able to do task independently, maximum 3 repeats **

---------------- 5 MINUTE BREAK ----------------

Bring the participant into the hallway for this section.

READING

Signs

"We will now practice reading a sign on the door. Like you did before, to read first feel with your hand where the item is. Put your head so that you are looking straight at the item. Take your hand away from the item. Tap your pointer finger ONCE on the raised line on the OrCam." *Guide trainee's hand and finger during instructions.*

"After tapping, you keep your head still and wait. The OrCam will go through the sounds you heard before. Then it will read or speak into your ear, so listen carefully."

"It is very important to face what you would like to read, to hold your head still, to tap the OrCam and to listen. Let's practice this!"

**Prompt with sign on door, adjust them as needed **

- "Please read the sign and tell me what it says."
- (Chime sound)→ "After you hear the chime, you can relax."
- "We will try this again."

** repeat until participant is able to do task independently, maximum 3 repeats **

FACE RECOGNITION

"We will now practice finding out who's in front of you using the OrCam. To find out who is in front of you, you tap your pointer finger ONCE on the raised line. I will guide your finger to show you." *Guide trainee's finger to tap on the ridged line towards the front of the camera.*

"After tapping, you keep your head still and wait. The OrCam will go through the sounds you heard before. Then it will speak into your ear, so listen carefully."

"It is very important to face the person, to hold your head still, to tap the OrCam and to listen. Let's practice this!"

***Prompt, adjust them as needed ***

- "Please find out and tell me who's in front of you."
- (Chime sound)→ "After you hear the chime, you can relax."
- "We will try this again."

** repeat until participant is able to do task independently, maximum 3 repeats **

Bring the participant back into the room for this part.

COLOR DETECTION

"You have been doing great job learning! We will now practice identifying the color of an item using the OrCam. This is the ONLY activity you will use pointing. To identify the color of an item, you hold your pointer finger out in front of you like a number one and

touch the item. I will guide your finger to show you." *Guide trainee's finger to have nailbed facing the OrCam.*

"After touching the item, you keep holding your finger there and wait. The OrCam will go through the sounds you heard before. Then it will speak into your ear, so listen carefully."

"It is very important to face the item, to hold your head still, and to hold your finger in front of you like a number one, and listen. Let's practice this!"

***Prompt with colored sheet, adjust them as needed ***

- "Please identify and tell me of the item in front of you."
- (Chime sound)→ "After you hear the chime, you can relax."
- "Let's try that again."

** repeat until participant is able to do task independently, maximum 3 repeats **

--------------- 5 MINUTE BREAK ----------------

iPad

"Now, I will show you how to read words on an iPad or computer screen using the OrCam. Like you did before, to read, you tap your pointer finger ONCE on the raised line. I will guide your finger to show you." *Guide trainee's finger to tap on the ridged line towards the front of the camera.*

"After tapping, you keep your head still and wait. The OrCam will go through the sounds you heard before. Then it will read or speak into your ear, so listen carefully."

"It is very important to face what you would like to read, to hold your head still, to tap the OrCam and to listen. Let's practice this!"

***Prompt with iPad, adjust them as needed ***

- "Please read and tell me what is on the screen."
- (Chime sound)→ "After you hear the chime, you can relax."
- "We will try this again."

** repeat until participant is able to do task independently, maximum 3 repeats **

RECOGNIZING SNACKS

"Now, I will show you how to read the words on snack packs using the OrCam. Like you did before, to read, you tap your pointer finger ONCE towards on the raised line. I will guide your finger to show you." *Guide trainee's finger to tap on the ridged line towards the front of the camera.*

"After tapping, you keep your head still and wait. The OrCam will go through the sounds you heard before. Then it will read or speak into your ear, so listen carefully."

"It is very important to face what you would like to read, to hold your head still, to tap the OrCam and to listen. Let's practice this!"

***Prompt with snacks, adjust them as needed ***

- "I will hold the snack in front of you"
- "Please read and thell me what is the snack in front of you."
- (Chime sound)→ "After you hear the chime, you can relax."
- "We will try this again."

** repeat until participant is able to do task independently, maximum 3 repeats **

TELLING TIME and DATE

"Now, I will show you how to find out the time and today's date. To do this, you hold your wrist in front of the OrCam. I will move your wrist to show you." *Guide trainee's wrist to the proper positioning.*

"While you hold your wrist, the OrCam will go through the sounds you heard before as it does what you asked. Then it will speak into your ear, so listen carefully."

"It is really important to hold your head still and keep holding your wrist even after it starts speaking."

**Prompt and adjust them as needed **

- "To tell the time and date, these are three things I want you to do place your wrist in front, hold, and listen.   Let's practice this."
- "Please find out and tell me the time and date."
- (Chime sound)→ "After you hear the chime, you can relax."
- "We will try this again."

** repeat until participant is able to do task independently, maximum 3 repeats **

TUTORIAL FOR PARENTS

Video tutorials ppt

Go over PowerPoint presentation of video tutorials.

Positioning of object

Demonstrate set-up using the stand.

1. Place the stand/reading material approximately 12 inches away and at eye level from where your child is seated.
2. Make sure that he/she is facing the material straight on.
3. Make sure there is adequate lighting.

OrCam Pairing to phone

1. Go to the app store and download ‘My Eye 2’
2. Follow the prompts on how to pair the My Eye
3. Place the device next to your phone
4. Position your device towards the ceiling
5. Tap ‘Pair’
6. Adjust the settings to the following:
    - ◊ Speed: 140 words/minute
    - ◊ Turn off bank note recognition
    - ◊ Turn off automatic features such as face recognition
    - ◊ Turn off voice-activated features
    - ◊ …
7. Go over the control panel

Face pairing

1. Position the parent ~3 feet away from the trainer wearing the glasses
2. The trainer will tap and hold, thus starting to learn the parent’s face
3. Prompt the parent to remove their face mask and turn their head to the right and left for 30 seconds.
4. Have the parent repeat their name when prompted by the OrCam.
5. Confirm the name by tapping.
