# Supplementary material for: Prospective pilot safety, feasibility study of an optic-to-audio device for children with CLN3 disease
Source: Orphanet J Rare Dis. 2026 Apr 3;21:199. doi: 10.1186/s13023-026-04319-0 (PMC13173725; doi:10.1186/s13023-026-04319-0)
Supplement: Supplementary file 4 — Supplementary Material 4: Additional File 6. Applicability questionnaire. [file 13023_2026_4319_MOESM4_ESM.pdf]

Study Participant ID ___________________________________

Date of Completion ___________________________________

Completed By Mother Father Other (specify) _____________________

APPENDIX E. Applicability Questionnaire

To be administered at baseline evaluation.

A.

Within the past month, please rate how often did your child encounter the activity.

0	1	2	3	4
Did not encounter	Encountered once	Encountered more than once, but less than weekly	Encountered weekly	Encountered daily

1. Recognize a school-related document. 0 1 2 3 4
2. Recognize options from a food Menu. 0 1 2 3 4
3. Recognize or read signs (e.g. sign for restroom, classroom, etc.) 0 1 2 3 4
4. Recognize a printed label (e.g. toothpaste, lotion, etc.) 0 1 2 3 4
5. Recognize text on a page in a book (e.g. snack packets, drawer, cupboard, etc.) 0 1 2 3 4
6. Recognize the color of an item (e.g. menus, books, etc.) 0 1 2 3 4
7. Recognize the face of a person 0 1 2 3 4
8. Report the day and date 0 1 2 3 4
9. Recognize packaged snack products 0 1 2 3 4
10. Recognize options in a Game Menu on an electronic device 0 1 2 3 4

Study Participant ID ________________________________

Date of Completion ________________________________

Completed By Mother Father Other (specify) ______________________

B.
Within the past month, please rate how helpful would a visual assistive device have been in helping your child to complete the specific activity.

0	1	2	3	4
Not helpful	Helpful	Helpful	Helpful	Helpful
	I would still have to help with 100% of the task	I would still have to help with up to 50% of the task	I would still have to help with up to 10% of the task	I would not have to help with the task

1. Recognize a school-related document. 0 1 2 3 4
2. Recognize options from a food Menu. 0 1 2 3 4
3. Recognize or read signs 0 1 2 3 4
   (e.g. sign for restroom, classroom, etc.)
4. Recognize a printed label 0 1 2 3 4
   (e.g. toothpaste, lotion, etc.)
5. Recognize text on a page in a book 0 1 2 3 4
   (e.g. snack packets, drawer, cupboard, etc.)
6. Recognize the color of an item 0 1 2 3 4
   (e.g. menus, books, etc.)
7. Recognize the face of a person 0 1 2 3 4
8. Report the day and date 0 1 2 3 4
9. Recognize packaged snack products 0 1 2 3 4
10. Recognize options in a Game Menu on an electronic device
    0 1 2 3 4

In the past month,

1) What daily function task(s) do you think your child would be able to do with minimal help if a device such as OrCam MyEye 2 was available?

2) What capability a device such as OrCam MyEye 2 should develop that would be more helpful for your child?
