# Supplementary material for: Prospective pilot safety, feasibility study of an optic-to-audio device for children with CLN3 disease
Source: Orphanet J Rare Dis. 2026 Apr 3;21:199. doi: 10.1186/s13023-026-04319-0 (PMC13173725; doi:10.1186/s13023-026-04319-0)
Supplement: Supplementary file 5 — Supplementary Material 5: Additional File 7. Ability questionnaire. [file 13023_2026_4319_MOESM5_ESM.pdf]

ADDITIONAL FILE 7

Study Participant ID ______________________________

Date of Completion ______________________________

Completed By Mother Father Other (specify) ____________________

APPENDIX D. Ability Questionnaire

To be administered at baseline, 1-week, and 1-month evaluations.

PURPOSE

We would like to understand your child's ability to do certain tasks relating to skills they may need to use in daily activities. We would like to understand how applicable do you think these skills would be for your child to have.

ABILITY

Within the past week, please rate your child's ability to do the task using the below scale.

0	1	2	3	4
Does not understand the task requested.	Understand the task requested.	Understand the task requested.	Understand the task requested.	Understand the task requested.
Unable to do	Unable to do without help for 100% of the task	Unable to do without help for 50% of the task	Unable to do without help for <5% of the task	Unable to do without help for <5% of the task
			Task completed appropriately <50% of the time	Task completed appropriately >90% of the time

1. Recognize a school-related document (e.g. homework assignment)
   0 1 2 3 4

2. Identify specific requested information on a school-related document
   0 1 2 3 4

3. Recognize options from a printed food Menu 0 1 2 3 4

4. Select a specific item from a food Menu	0	1	2	3	4
5. Recognize a room sign	0	1	2	3	4
6. Identify the appropriate restroom sign for entry	0	1	2	3	4
7. Recognize printed labels (e.g. for drawers, objects)	0	1	2	3	4
8. Identify specifically requested label	0	1	2	3	4
9. Recognize text on a book page	0	1	2	3	4
10. Identify answer to specific question about the information in the book page	0	1	2	3	4
11. Recognize the color of an item	0	1	2	3	4
12. Recognize a face	0	1	2	3	4
13. Report the day and date	0	1	2	3	4
14. Recognize a packaged food product	0	1	2	3	4
15. Recognize options from a game menu from an electronic device	0	1	2	3	4
