# Supplementary material for: Prospective pilot safety, feasibility study of an optic-to-audio device for children with CLN3 disease
Source: Orphanet J Rare Dis. 2026 Apr 3;21:199. doi: 10.1186/s13023-026-04319-0 (PMC13173725; doi:10.1186/s13023-026-04319-0)
Supplement: Supplementary file 6 — Supplementary Material 6: Additional File 8. Feasibility questionnaire. [file 13023_2026_4319_MOESM6_ESM.pdf]

ADDITIONAL FILE 8

Study Participant ID ____________________________________

Date of Completion ____________________________________

Completed By Mother Father Other (specify) ______________________

APPENDIX B. Feasibility Questionnaire

To be done at 1-week and 1-month evaluations.
Scoring: No = 0; Yes = 1

Please answer the below items.

1. The device's user instruction is simple to follow. No Yes
   If No, please indicate how the instruction can be made to be more understandable.
   a. in-person, on-demand user support available
   b. more speech commands (rather than touch/swipe) to select options
   c. other, please specify
2. The device's reading function is easy to use. No Yes
   If No, please indicate how the function can be made to be easier to use
   a. less dependence on background lighting
   b. less restriction on field of image captured/object positioning
   c. more variety of recognized text fonts
   d. better recognition of column/page spacing boundaries
   e. better discrimination between text and non-text
   f. other, please specify
3. The device's face recognition function is easy to use . No Yes
   If No, please indicate how the function can be made to be easier to use
   a. more speech commands to select options
   b. less restriction on field of image captured/object positioning
   c. other, please specify
4. The device's color identification is easy to use. No Yes
   If no, please indicate how the function can be made to be easier to use
   a. less restriction on field of image captured/object positioning
   b. more accurate color identification
   c. other, please specify
5. The device is easy for my child to use around the house. No Yes
   If No, please indicate how the device can be made to be easier to use
   a. more speech commands using natural speech (e.g. turning device on/off)

b. more secure device attachment
c. more variety of recognized text fonts
d. other, please specify

Study Participant ID ________________________________

Date of Completion ________________________________

Completed By Mother Father Other (specify) ______________________

6. The device is easy for my child to use outside of the house. No Yes
   If no, please indicate how the device can be made to be easier to use
   a. better battery life
   b. more secure device mounting
   c. more variety of recognized fonts
   d. other, please specify
7. My child asked to use the device. No Yes
8. My child used the device without needing my help. No Yes
9. The device may be a useful visual assistive tool for individuals with CLN3-Batten in daily life.
   No Yes
10. If available, my child with CLN3-Batten would use the device in his/her daily life.
    No Yes
11. I would recommend the device to other individuals with CLN3-Batten.
    No Yes
