# Supplementary material for: Prospective pilot safety, feasibility study of an optic-to-audio device for children with CLN3 disease
Source: Orphanet J Rare Dis. 2026 Apr 3;21:199. doi: 10.1186/s13023-026-04319-0 (PMC13173725; doi:10.1186/s13023-026-04319-0)
Supplement: Supplementary file 7 — Supplementary Material 7: Additional File 4. Optional Day 33 study extension. Additional File 5. OrCam MyEye 2. Additional File 9. Parental choice of signs or symptoms of CLN3 disease they would like to see being addressed by future interventions. Additional File 10. PedEyeQ scaled scores of parental perspectives at baseline visit. Additional File 11. Evaluation of feasibility of using the device by parental questionnaire at visit Day 5. Additional File 12. Time-to-task completion of Function Test items. [file 13023_2026_4319_MOESM7_ESM.docx]

**Prospective Pilot Safety, Feasibility Study of an Optic-To-Audio Device for Children with CLN3 Disease**

**A)**

**
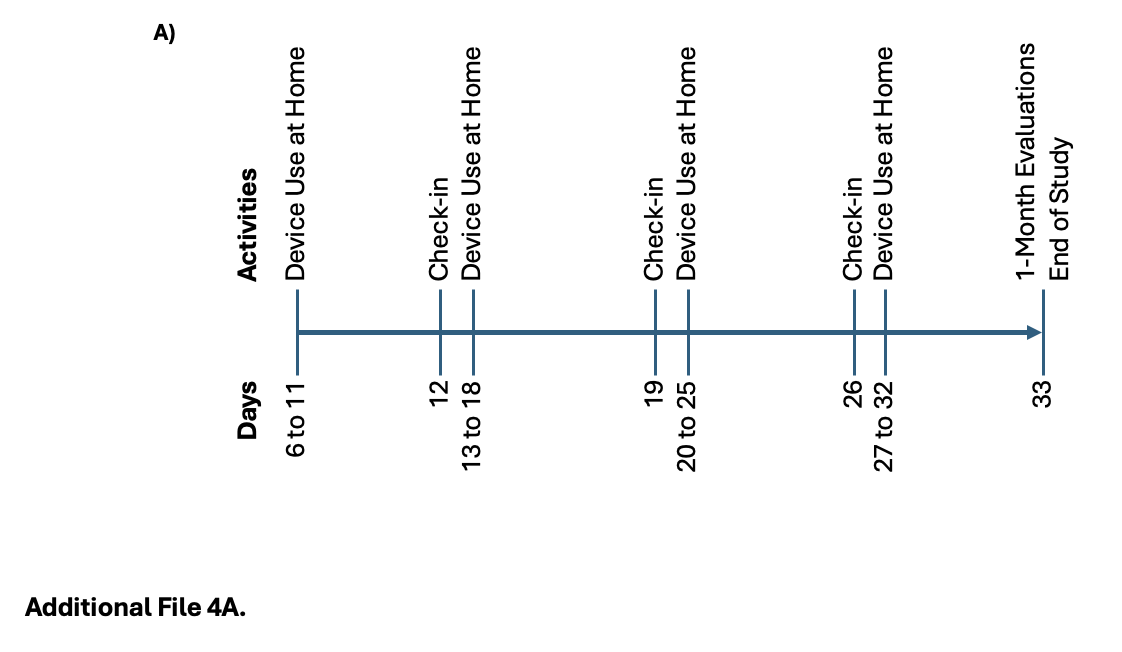
**

**B)**

**
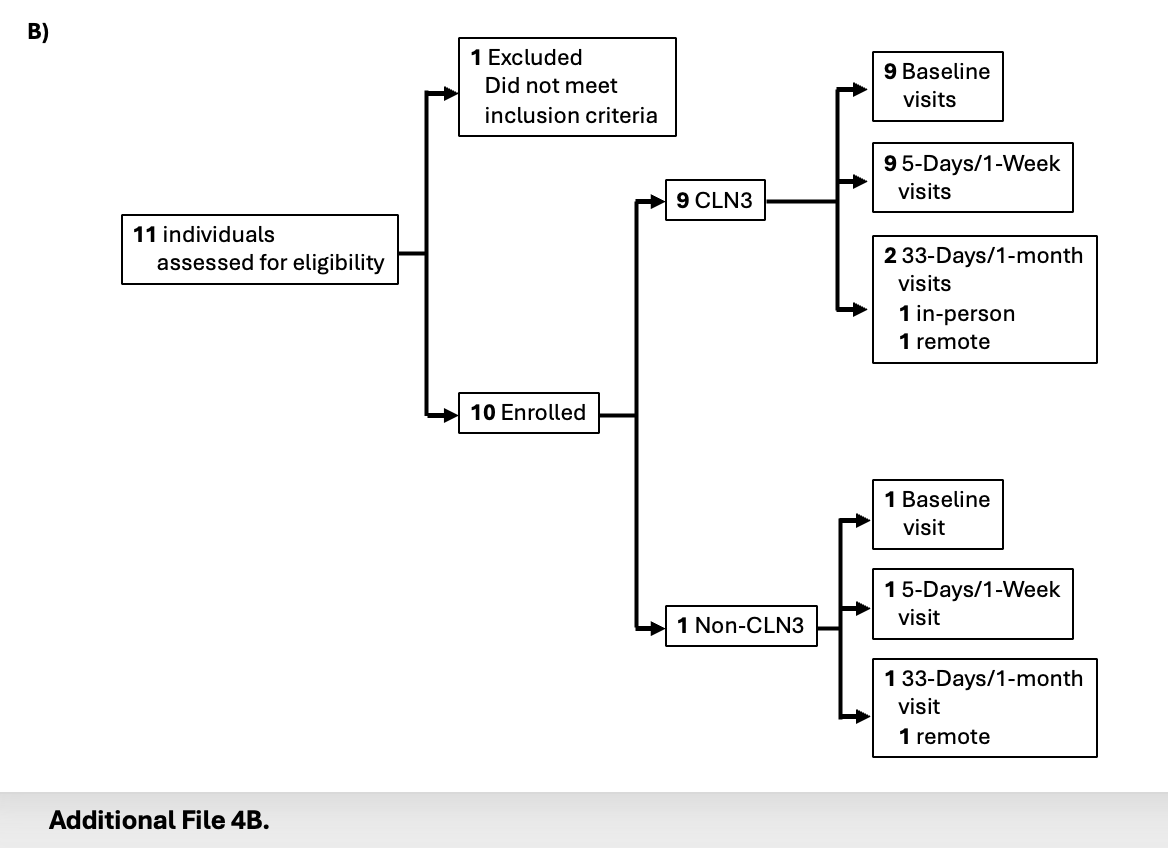
**

**Additional File 4. Optional Day 33 study extension.** **A)** Study schema **B)** Participant enrollment and evaluations.


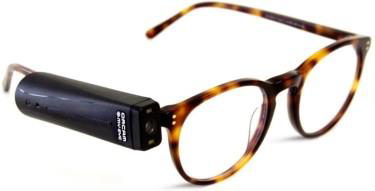


**Additional File 5. OrCam MyEye 2.** https://www.orcam.com/en/blog/category/general/. Accessed March 8, 2020.


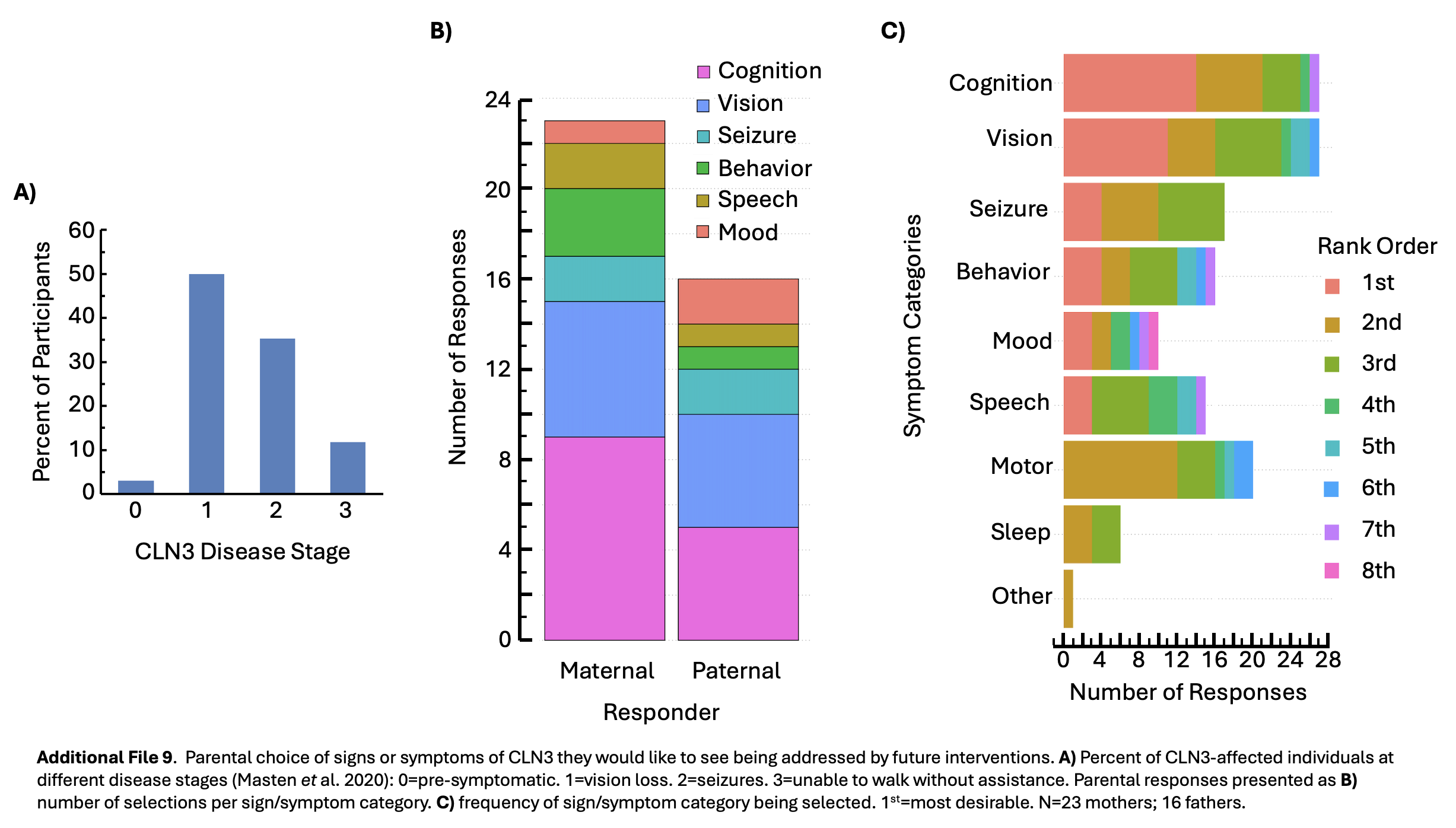


**Additional File 9.** **Parental choice of signs or symptoms of CLN3 they would like to see being addressed by future interventions**. **A)** Percent of CLN3-affected individuals at different disease stages (Masten MC *et al*. 2020): 0=pre-symptomatic. 1=vision loss. 2=seizures. 3=unable to walk without assistance. Parental responses presented as **B)** number of selections per sign/symptom category. **C)** frequency of sign/symptom category being selected. 1st=most desirable. N=23 mothers; 16 fathers.


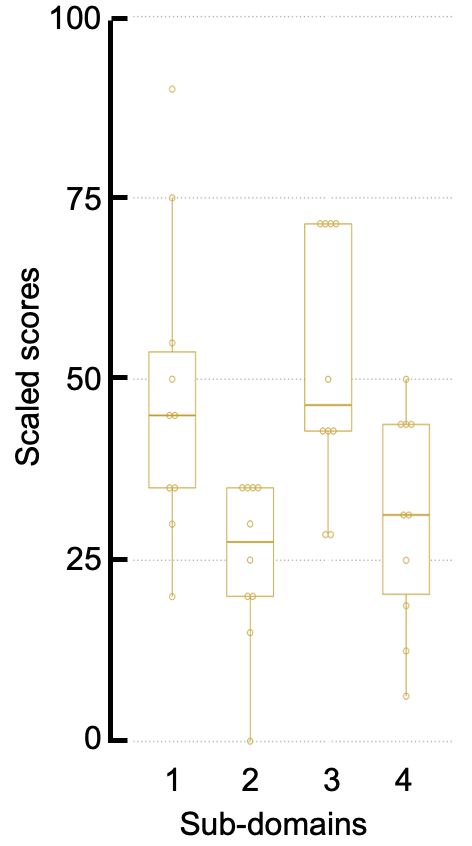


**Additional File 10. PedEyeQ scaled scores of parental perspectives at baseline visit.** Sub-domain 1=impact on family. 2=worry about eye condition. 3=worry about interactions. 4=eyecare. Horizontal bar: median. Box: interquartile range. Whiskers: 1.5x IQR.

**Additional File 11. Evaluation of feasibility of using the device by parental questionnaire at visit Day 5.**

| **Test Items** | **Number of Responses (CLN3/non-CLN3), n** | | |
| --- | --- | --- | --- |
|  | **Captured** | **Yes** | **No** |
| 1. Device’s user instruction is simple to follow. | 9/1 | 9/1 | 0/0 |
| 2. Device’s reading function is easy to use. | 9/1 | 6/1 | 3/0 |
| 3. Devices’ face recognition function is easy to use. | 9/1 | 9/1 | 0/0 |
| 4. Device’s color identification is easy to use. | 9/1 | 5/1 | 4/0 |
| 5. Device is easy for my child to use around the house. | 9/1 | 8/1 | 1/0 |
| 6. Device is easy for my child to use outside of the house. | 8/1 | 2/1 | 6/0 |
| 7. My child asked to use the device. | 9/1 | 6/1 | 3/0 |
| 8. My child used the device without needing my help. | 9/1 | 7/1 | 2/0 |
| 9. Device may be a useful assistive tool for individuals with CLN3 in daily life. | 9/0 | 9/0 | 0/0 |
| 10. If available, my child would use the device in his/her daily life. | 9/0 | 6/0 | 3/0 |
| 11. I would recommend the device to other individuals with CLN3. | 9/0 | 7/0 | 2/0 |

**Additional File 12**. Time-to-task completion of Function Test items at Day 1 and Day 5.

| Test Items | Visit Day | Number of Participants | Time to complete task (seconds) | |
| --- | --- | --- | --- | --- |
|  |  |  | Median | IQR |
| 1A | D01 | 9 | 180.0 | 0.0 |
| 1A | D05 | 9 | 14.9 | 22.6 |
| 1B | D01 | 9 | 180.0 | 0.0 |
| 1B | D05 | 9 | 3.1 | 1.0 |
| 2A | D01 | 9 | 180.0 | 0.0 |
| 2A | D05 | 9 | 19.0 | 8.5 |
| 2B | D01 | 9 | 180.0 | 0.0 |
| 2B | D05 | 9 | 15.5 | 30.9 |
| 3A | D01 | 9 | 180.0 | 39.8 |
| 3A | D05 | 9 | 8.0 | 9.4 |
| 3B | D01 | 9 | 180.0 | 131.8 |
| 3B | D05 | 9 | 5.1 | 25.3 |
| 4A | D01 | 9 | 180.0 | 0.0 |
| 4A | D05 | 9 | 5.1 | 4.2 |
| 4B | D01 | 9 | 180.0 | 0.0 |
| 4B | D05 | 9 | 3.8 | 27.0 |
| 5A | D01 | 9 | 180.0 | 0.0 |
| 5A | D05 | 9 | 27.3 | 12.7 |
| 5B | D01 | 9 | 180.0 | 0.0 |
| 5B | D05 | 9 | 26.3 | 20.7 |
| 6A | D01 | 9 | 19.8 | 178.1 |
| 6A | D05 | 9 | 12.2 | 16.1 |
| 6B | D01 | 9 | 7.8 | 136.6 |
| 6B | D05 | 9 | 2.7 | 2.0 |
| 7A | D01 | 9 | 180.0 | 169.9 |
| 7A | D05 | 9 | 5.2 | 10.3 |
| 7B | D01 | 9 | 180.0 | 167.4 |
| 7B | D05 | 9 | 5.1 | 12.5 |

D01: Day 1. D05: Day 5. IQR: interquartile range.
